# Structural characterization of the extracellular stalk material of the diatom *Didymosphenia geminata*

**DOI:** 10.1007/s00216-024-05370-1

**Published:** 2024-06-10

**Authors:** Lara Dütsch, Erica Brendler, Jan Zuber, Christine Viehweger, Hermann Ehrlich, Teofil Jesionowski, Carla Vogt

**Affiliations:** 1 Institute of Analytical Chemistry, TU Bergakademie Freiberg, Leipziger Str. 29, 09599 Freiberg, Germany; 2 Institute of Geology, TU Bergakademie Freiberg, Gustav-Zeuner-Str. 12, 09599 Freiberg, Germany; 3 Center for Advanced Technology, Adam Mickiewicz University, Uniwersytetu Poznańskiego 10, 61-614 Poznań, Poland; 4 Institute of Chemical Technology, Faculty of Chemical Technology, Poznań University of Technology, Berdychowo 4, 60-965 Poznań, Poland

**Keywords:** *Didymosphenia geminata*, Lignin, HR-MS, NMR spectroscopy, Fluorescence microscopy, Extracellular polymeric substances

## Abstract

The study represents new bioanalytical characterization of mainly organic components of the poorly investigated extracellular polymeric substances (EPS) of the enigmatic diatom *Didymosphenia geminata*, an invasive, worldwide expanding species endangering diverse ecosystems. This microalga attaches its siliceous cells to rocky substrates using fibrous stalks, which are made of an EPS-based matrix stabilized by crystalline calcite. The EPS were analyzed using selected methods, including microscopic, spectroscopic, and spectrometric techniques. We identified diverse types of biomolecules. The presence of lipids, condensed aromatics, and heteroaromatic compounds in the EPS has been confirmed using high-resolution mass spectrometry (HR-MS). Additionally, both sulfur-containing functionalities and carboxylic acids were determined too using infrared (IR) spectroscopy and nuclear magnetic resonance (NMR) spectroscopy. For the first time, lignin compounds have been detected as one of the components of the EPS of the *D. geminata* diatom, using HR-MS and fluorescence microscopy (FM) in combination with specific staining techniques. By increasing the understanding of the chemistry and structural features of the stalks, we aim to develop potential applications and methods for removing these stalks from affected regions in the future, or, alternatively, to use them as a large-scale source of sustainable biocomposite material.

**Graphical abstract:**

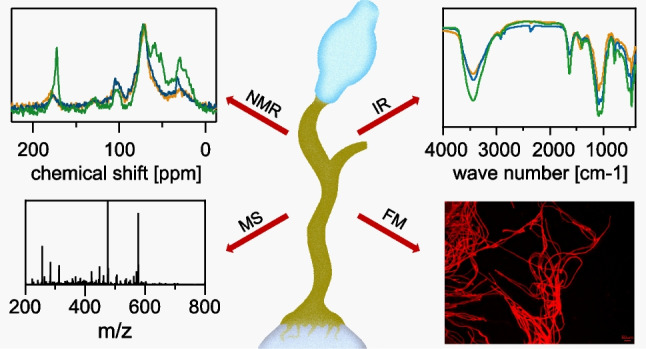

**Supplementary Information:**

The online version contains supplementary material available at 10.1007/s00216-024-05370-1.

## Introduction

*Didymosphenia geminata* (Lyngb.) M. Schmidt 1899, also known as Didymo [1], is an invasive diatom species present in cool-water niches (i.e., rivers) [2]. The species was first discovered in the nineteenth century by H. C. Lyngbye on the Faroes Islands in Northern Europe [3]. Due to globalization, the microalgae have spread worldwide [2]. A distinctive feature of this species is its cell body made of amorphous silica (SiO_2_) [4]. The apical pore of the frustule emits an extracellular polymeric substance (EPS) [5] in the form of a stalk. The stalks then branch through the division of the cells, leading to the formation of vast networks [6]. Especially oligotrophic conditions in combination with high light intensities lead to the formation of longer stalks [7]. This phenomenon is known as the diatoms bloom. The mats, consisting mainly of EPS, can have a thickness of up to 20 cm and adversely affect the ecosystem [8]. Because sediments can be trapped in the network, the benthic surface is altered. This has an effect on the organisms present in the water body. Various studies have demonstrated that with an increase in stalk content, a modification in the invertebrate density takes place [9–11].

In general, oligotrophic conditions with low concentrations of soluble reactive phosphorus promote the synthesis of EPS [12–14]. Studies have shown that an increasing phosphorous concentration leads to an increase in the frequency of dividing cells (FDC) and hence shorter stalks [7]. Additionally, a higher light intensity influences the cell division rate resulting in longer stalks, as demonstrated by various experiments by Kilroy and Bothwell [7, 15, 16]. The production of EPS in nutrient-poor rivers is due to a “photosynthetic overflow.” As a result of the lack of phosphorus, cell division is inhibited and the excess carbon produced by photosynthesis is released as EPS [7]. This phenomenon has been previously studied also in other diatoms [17–19].

Analyses of the stalks were previously focused on selected parts of the EPS. Organic components were mainly investigated with regard to the composition of the polysaccharide matrix. In previous studies, it has been reported that the stalks of Didymo consist of a polysaccharide mainly composed of galactose (Gal) and xylose (Xyl) linked by 3,4-Gal and 4-Xyl [20]. The polysaccharide is sulfonated [20], as it was confirmed by IR spectroscopy [21]. The monosaccharide composition was verified by Figueroa et al. [21] through acidic hydrolysis of the polysaccharide and identification using high-performance thin-layer chromatography (HPTLC). By applying different extraction protocols, the overall content of the substance classes of lipids and proteins has been determined [21]. The elemental composition has been published before using an elemental analyzer [21] and energy-disperse X-ray spectroscopy (EDX) [4].

The surface of the stalks produced by Didymo is vertically striated, as can be seen on corresponding scanning electron microscopy (SEM) images represented before [4, 22, 23]. Three layers could be identified by preparing cross-sections and analyzing them using transmission electron microscopy (TEM) [22, 23]. Calcium deposits have been also identified in the stalks through the combination of SEM and EDX [4]. This was the first report on calcification in biosilica-producing diatoms. The calcium is present in the form of nanocrystalline calcite (CaCO_3_) and has been confirmed by electron diffraction and fast Fourier transformation (FFT) of high-resolution transmission electron micrographs (HRTEM) as well as X-ray diffraction (XRD) [4]. Crystalline calcite nanofibers are emitted from the apical pores of siliceous cells and have a diameter of approximately 170 nm [4]. According to Ehrlich et al. [4], calcite provides additional mechanical stability to elevate the cells further for better access to light. In addition, it can act as a reservoir for ions and small molecules [4]. Furthermore, Aboal et al. [23] proposed that elevating the cells enhances both gas exchange and nutrient uptake, particularly with regard to phosphate.

Further insights into the structural composition of EPS are needed to develop potential application (i.e., that on large scale) for the stalks or to find key ways to remove the stalks that are resistant to degradation from ecosystems [8]. Thus, according to the modern view [4], the stalks of *D. geminata* represent an example of a highly structured, microtubular organic-inorganic biocomposite, where the chemistry of the organic part includes several open questions. Consequently, the purpose of this study was to characterize organic components of the EPS of Didymo using a battery of analytical tools including high-resolution mass spectrometry (HR-MS), fluorescence microscopy, infrared (IR) spectroscopy, and solid-state nuclear magnetic resonance (NMR) spectroscopy.

IR spectroscopy and solid-state ^13^C-NMR spectroscopy are useful techniques for a rapid, easy analysis of a sample with focus on its main components, here the polysaccharide matrix, as well as information about the differences between the investigated samples is obtained. Using solid-state NMR, signals corresponding to aromatic moieties in the EPS are detected that lead to further investigations using HR-MS and fluorescence microscopy. Fourier transform ion cyclotron resonance (FT-ICR) mass spectrometry in combination with graphite-assisted laser desorption/ionization (GALDI) proved to be suitable for the analysis of biomaterials as previous studies showed [24, 25]. High polar as well as less polar compounds can also be analyzed with the help of this method, as shown for a lignin reference [24]. A comprehensive evaluation of various biomolecules can be carried out through an analysis of the molecular formula lists. These analyses provide insight into the heteroatomic class distribution. By plotting the number of carbon atoms (*n*_*C*_) against the double bond equivalent (DBE), one is able to distinguish aromatic moieties from carbohydrates or lipids. Also, van Krevelen plots can be utilized to visualize different classes of biomolecules, like condensed aromatic compounds, lignin and lignin-like oligomers, lipids, or carbohydrates. Both the *n*_*C*_-DBE plots and van Krevelen plots confirmed that lignin-like molecules are a component of the EPS. Additionally, fluorescence microscopy combined with the staining of a sample is a great tool for identifying lignified tissues and fibers. Thus, using Safranin O and Acridine Orange as appropriate dyes, the presence of lignin was further verified. In conclusion, our findings indicate that we are the first to identify lignin-like molecules in the EPS of a diatom species.

## Methods and materials

Samples of the diatom *D. geminata*, purchased from INTIB GmbH Freiberg, were collected from the river San in Poland and were processed as described in [4]. The *D. geminata* specimens under study were identified by Prof. Andrzej Witkowski, Szczecin University, Poland, in 2016. The diatoms and their stalks were scraped from the rocks, washed in distilled water, and collected. In the following step, siliceous cells and stalk material were separated by ultrasonic treatment in a nylon bag (mesh size 160 µm). 6 M hydrochloric acid was used to demineralize the sample by removing the calcite matrix. In the last step, the demineralized stalks were extracted with a 1 M EDTA solution to eliminate paramagnetic components such as iron ions, as well as cations such as magnesium and calcium. From these treatments, three different samples for further investigations resulted: untreated stalks (Stalk_raw), demineralized stalks (Stalk_HCl), and demineralized and with EDTA extracted stalks (Stalk_EDTA).

### IR spectroscopy

Potassium bromide (KBr, purchased from *Carl Roth GmbH*, for IR spectroscopy) discs (11 mm) for all three samples were prepared by milling approximately 300 mg KBr with 0.5 mg of the Didymo sample in an Ardenne ball mill (by *VEB Narva*, ball diameter 1 cm) for 10 min, filled into the pressing tool (hydraulic pellet press by *Specac Ltd*), and the mixture was pressed to pellets with a pressure of 9 t for 2 min. The analyses of the pellets placed in a sample holder were conducted in the transmission mode using a *Nicolet iS10* FT-IR spectrometer by *Thermo Fisher Scientific*. The software *OMNIC* version 9.8.372 was used for the recording process. A background scan recorded before collecting sample data was subtracted from the sample spectrum. For background and sample scans, 32 scans were recorded, respectively. The spectra were further visualized using *OriginPro 2019b*.

### Solid-state NMR

Solid-state NMR spectra were recorded on an *AVANCE III HD 400 MHz WB* (wide bore) NMR spectrometer from *Bruker BioSpin GmbH*. A 4 mm *MAS DVT 400 WB triple resonance* probe was used with 4 mm zirconium oxide rotors and polychlorotrifluoroethylene caps. Spectra were recorded at room temperature using magic angle spinning (MAS) at 10 kHz to average the anisotropic interactions to zero. The samples were investigated using a single pulse excitation (SP, pulse program: hpdec) and cross-polarization (CP, pulse program: cp). For the ^13^C-CP/MAS-NMR spectra, a relaxation delay of 2 s, a contact time of 1 ms, and 30 k scans were used. To achieve the complete relaxation of crystalline calcite in the Stalk_raw sample, the delay time for the ^13^C-SP/MAS-NMR analysis was set to 60 s and 4 k scans were recorded. The demineralized sample, Stalk_HCl, could be analyzed using a shorter delay time of 1 s and more scans (80 k) for the SP/MAS-NMR spectrum because of the lack of calcite and the presence of paramagnetic cations. Raw data were collected and processed using *TopSpin* (version 3.6.2) by *Bruker BioSpin GmbH*. Additionally, *MestReNova* (version 14.1.0-24037) by *Mestrelab Research* was used for further visualization of the data.

### FT-ICR-mass spectrometry

The Stalk_raw sample was investigated using positive and negative ion mode GALDI-MS. A 10 g/l stock solution of the stalks in methanol (by *Supelco*, hypergrade for LC-MS) was prepared and sonicated for 10 min. The sample was allowed to sediment and 200 µl of the supernatant was combined with 30 mg of ultrapure graphite (by *Micro to Nano*, purity ≥ 99.5%, particle size 5 µm), which was weighed beforehand in a glass vial. The solution was then sonicated for additional 10 min. Next, the suspension was applied to a stainless-steel target (spot volume 1.2 µl).

All measurements were performed on a *15 T solariX* FT-ICR mass spectrometer by *Bruker Daltonics*, equipped with an LDI (laser desorption/ionization) source (Smart Beam II laser, frequency tripled Nd:YAG laser, *λ* = 355 nm, pulse duration 3 ns, pulse energy 500 µJ, peak power 170 kW, average power 1.5 W). The GALDI-MS analysis required a minimum laser focus, a laser power of 45% (GALDI(−)) or 55% (GALDI(+)), a laser shot number of 20, and a laser frequency of 500 Hz. A scan range of 153.51–2000.00 Da was used. Each single measurement was performed by accumulating 64 scans using the software *ftmsControl 2.2*. A resolution *R* of 800,000 was determined at *m/z* = 400 Da for all analyses.

*DataAnalysis 5.0* from *Bruker Daltonics* and *MATLAB R2022b* were used to analyze the results. The mass spectra were calibrated using internal calibration lists. *DataAnalysis* was used to calculate the molecular formulae of carbon clusters. The generated internal calibration lists were then applied to the spectra. The molecular formulae for peaks with a signal-to-noise ratio (SNR) > 10 were calculated using the following elemental compositions: C_*c*_H_*h*_N_*n*_O_*o*_S_*s*_: *c* = unlimited, *h* = unlimited, 0 ≤ *n* ≤ 3, *o* = unlimited, 0 ≤ *s* ≤ 5. The peak lists and molecular formula lists were imported into *MATLAB* and further processed using in-house scripts. First, the data were blank-corrected and filtered. The criteria for the filtering of molecular formula lists followed the rules recommended by Herzsprung [26, 27]: double bond equivalent (DBE) ≥ 0, 0.3 ≤ *H/C* ≤ 2.5, *O/C* ≤ 1.0, *N/C* ≤ 1.0, *S/C* ≤ 1.0. The blank-corrected and filtered data were then further visualized and evaluated.

### Fluorescence microscopy

The samples defined as Stalk_raw and Stalk_EDTA as well as a lignin standard (Indulin) were stained with two different fluorescence markers. A solution of 0.2% *w/v* Safranin O (SO) in 50% ethanol *w/v* and two different concentrations of Acridine Orange (AO) in water (0.01 M and 10^−6^ M) were used. For each sample, 3–7 mg of the stalk material were stained in 50 ml tubes with 20 ml of the staining solution. After 30 min, the tubes were centrifuged for 5 min (5000 rpm), decanted, and washed with 20 ml distilled water. The procedure was repeated twice. Finally, the stained stalk material was washed with another 10 ml of distilled water three times before drying the material. Fluorescence microscopy images were obtained using a *Keyence BZ-9000* digital optical microscope (*Keyence*, Osaka, Japan) with zoom lenses CFI Plan Apo 10 × and CFI Plan Apo 40 × , using the GFP channel (Ex/Em = 470/525) for green-stained samples, and the TxRed channel (Ex/Em = 560/630) for red-stained samples, and the bright field for comparison. The light exposure time was adapted for each sample individually.

## Results and discussion

### IR spectroscopy

**Fig. 1:**
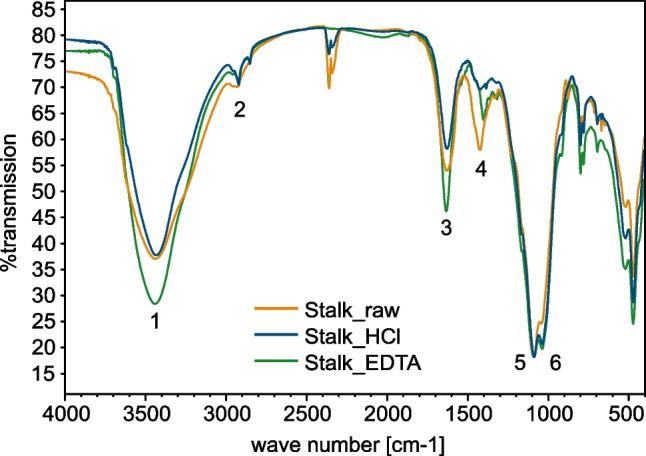
IR spectra of all three Didymo samples, prepared as KBr disks, normalized to the most intense signal (1088 cm^−1^), signals at a wave number of 2360 cm^−1^ are C=O stretching vibrations of carbon dioxide

IR spectra of all three samples were prepared as described above and recorded as KBr disks. The spectra have been normalized to the most intense signal at a wave number of 1088 cm^−1^, as can be seen in Fig. 1. The broad strong signal at 3450 cm^−1^ can be assigned to O-H stretching vibrations of hydroxy groups (band 1). The smaller signal below 3000 cm^−1^ is characteristic for C-H stretching vibrations in aliphatic CH, CH_2_, and CH_3_ groups (band 2). The sharp signal at 1630 cm^−1^ was assigned to the C=O stretching vibration of carbonyls (band 3). C-H bending vibrations of aliphatic groups were assigned to the band at 1423 cm^−1^ (band 4). The two sharp signals at 1090 cm^−1^ and 1035 cm^−1^ are derived from C-O stretching vibrations of hydroxy groups as well as S=O stretching vibrations of sulfoxides and sulfonic acids (bands 5 and 6). The broad band 1 indicates a significant amount of hydroxy groups and water present in the sample [28].

Hydroxy groups are characteristic for carbohydrates as well as for lignin (phenolic OH). Previous investigations revealed that the primary component of the stalk is a carbohydrate matrix, which explains the high amount of hydroxy groups [20]. Additionally, the green curve for the sample Stalk_EDTA showed slightly higher values for bands 1 and 3. The primary reason for this increase is the presence of remaining EDTA, which could not be completely removed from the sample despite the extensive washing process. The vibrations of aromatic compounds are not visible due to the overlapping of the O-H stretching vibration with the region that is typical for aromatic C-H stretching vibrations (3100 to 3000 cm^−1^) [28]. S=O stretching vibrations (band 6) have been detected in IR spectra before [21]. Previous studies have demonstrated that S-containing groups have the ability to adsorb various metal cations [29, 30]. In general, the IR spectra reported by Figueroa et al. [21], who investigated samples from Chile, are comparable to the spectra measured here with differences primarily observed in the fingerprint region below 1500 cm^−1^.

### Solid-state NMR spectroscopy

^13^C-MAS-NMR spectroscopy was applied to characterize the main components of the stalks. Both direct excitation (singe pulse, SP) and cross-polarization (CP) spectra were recorded. In general, five main regions can be distinguished. Region 1 shows signals of carbonyls at a chemical shift δ of 190 to 160 ppm. An additional signal at 168 ppm is observed for the sample Stalk_raw when compared with the HCl-treated samples, marked by a black arrow. The signal indicates the presence of carbonates, in the form of crystalline calcite (CaCO_3_). In region 2 (150 to 110 ppm), signals of aromatic components are detected. Except for the sample Stalk_EDTA, the concentration of aromatic compounds is too low to yield detectable signals in this region. The signal at 100 ppm is attributed to the C_1_ atom of carbohydrates, also known as anomeric carbon [31] (signal 3). The signals at 70 ppm are assigned to C_2_ to C_5_ of carbohydrates (region 4). The C_6_ atom of carbohydrates has even lower chemical shift values around 60 ppm [31]. Signals of the C_6_ atom of hexoses are thus located on the flank of the broad signal at 70 ppm. Chemical shifts below 50 ppm indicate aliphatic groups that include CH, CH_2_, and CH_3_ (region 5).

**Fig. 2:**
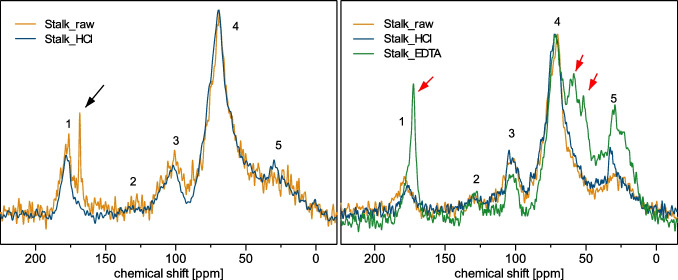
Left: ^13^C-SP/MAS-NMR spectra of Stalk_raw and Stalk_HCl; right: ^13^C-CP/MAS-NMR spectra of all three samples; black arrow: crystalline calcite; red arrows: EDTA; spectra scaled to equal intensity for the carbohydrate signal (4) at about 70 ppm

Figure 2 (right) displays the ^13^C-CP/MAS-NMR spectra of all three samples under study. A comparison of the CP- and SP spectra shows a noticeable similarity for both NMR analyses. In the ^13^C-CP/MAS-NMR spectra, only the sample Stalk_EDTA showed differences compared to the other two samples. Extracting the demineralized stalks with an aqueous solution of EDTA removed paramagnetic components, primarily transition metal cations, which prevented an efficient CP transfer due to accelerated proton relaxation. However, EDTA could not be removed completely from the sample, which results in additional signals in the spectrum, indicated by red arrows (Fig. 2, right). The high content of deprotonated carboxylic acid groups in EDTA explains the increase and shift of the signal from 177 to 172.6 ppm. The signals at 61.2 ppm, 58.8 ppm, and 51.9 ppm can be assigned to the acetate CH_2_ and ethylenic CH_2_, respectively [32]. By extracting the Stalk_EDTA sample with distilled water, at least 50% of the EDTA could be removed. The removal led to an increase in signal intensity in regions 2 and 5. A comparison of the CP/MAS-NMR spectra before and after extraction and all individual spectra are shown in the Supporting Information S1 together with a list of the assignments for the most important regions and signals. To gain additional information about the content of paramagnetic components, electron paramagnetic resonance (EPR) spectra of all samples were recorded (see SI S2, Fig. S7-S9). Information about the EPR spectrometer and the parameters used are summarized in SI S2; however, the results are not the focus of this article.

### FT-ICR-MS

HR-MS is a powerful analytical technique that provides valuable insights into the structural diversity of a sample. Previous research has demonstrated that GALDI is highly efficient for ionizing wood and plant samples that contain lignin compounds [24]. Therefore, we applied GALDI-MS as an ionization technique for the analysis of the Didymo stalks to identify components beyond the carbohydrate and calcite matrix. For this purpose, only the untreated stalks were analyzed in both positive and negative ion modes to gain first insights into the structural diversity.

**Fig. 3:**
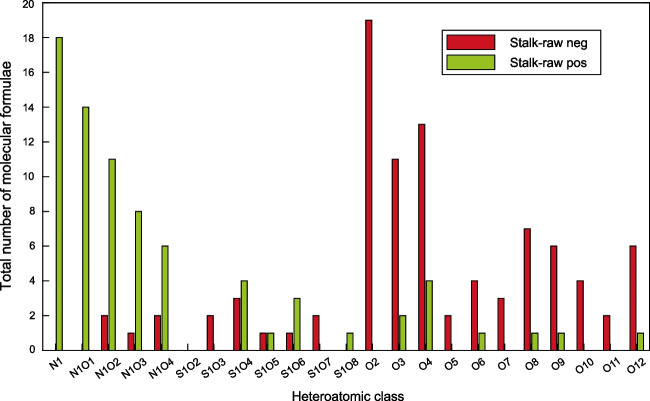
Total number of molecular formulae in different heteroatomic classes for Stalk_raw, GALDI(−)- (red) and GALDI(+)-MS (green)

In general, in the negative ion mode 2397 peaks and in the positive ion mode 2606 peaks were detected (mass spectra in SI S3, Fig. S10). Molecular formulae of the peaks based on their *m/z* were calculated for peaks with an SNR > 10 and further classified by their heteroatomic numbers. The absolute number of molecular formulae for the most interesting heteroatomic classes is provided in Fig. 3. The classes N_1_, N_1_O_1_ to N_1_O_4_, S_1_O_2_ to S_1_O_8_ and O_2_ to O_12_ were chosen here, because the highest number of molecular formulae in each mode was detected for these classes.

Certain classes of biomolecules are preferentially ionized depending on the ionization mode used. In the positive ion mode (green bars), (basic) nitrogen-containing molecules are more easily detected. The analyses showed that compounds with lower oxygen numbers (classes O_2_ to O_4_) are better ionized in the negative ion mode. Such structures can comprise carboxylic acid groups that easily deprotonate and form negatively charged ions. Furthermore, molecular formulae with a higher oxygen content are particularly detected in the negative ion mode. Examples of biomolecules with higher oxygen contents include carbohydrates and lignin. Hydroxy groups are easily deprotonated, making them detectable in the negative ion mode. In addition to the absolute number of formulae in each heteroatomic class, its relative abundance can also be plotted (see SI S4, Fig. S11). The relative abundance is influenced by the intensity of each peak and is therefore higher for heteroatomic classes with ions that show higher intensities in the mass spectrum.

**Fig. 4:**
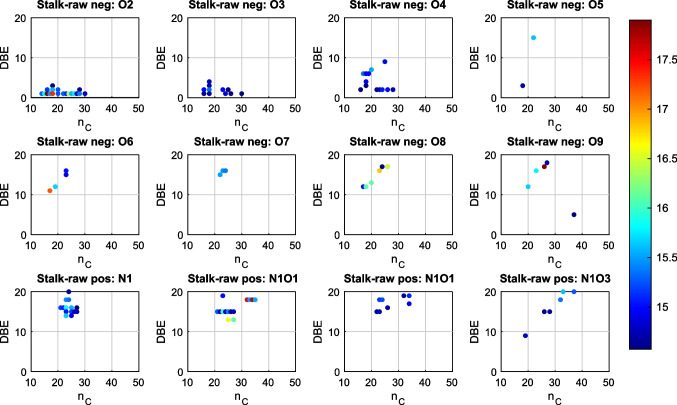
*n*_*C*_-DBE plots for selected heteroatomic classes O_2_ to O_9_ (GALDI(−)) and N_1_ and N_1_O_1_ to N_1_O_3_ (GALDI(+)), the log(intensity) of the DBE values are presented color-coded in a range of 14.5–18

To gain further insights into the structural diversity of each dataset, the number of carbon atoms (*n*_*C*_) of a molecular formula was plotted against its double bond equivalent (DBE) in *n*_*C*_-DBE plots (Fig. 4 for compound classes O_2_ to O_9_ in the negative mode, N_1_ and N_1_O_1_ to N_1_O_3_ in the positive mode). In section S5 in the SI (Fig. S14 and S15), all *n*_*C*_-DBE plots of the mentioned heteroatomic classes in Fig. 3 are summarized. This type of plot provides information regarding the molecular size of each ion based on its heteroatomic number and DBE values. In the GALDI(−)-MS analyses, most compounds in the O_2_ class have a DBE of one and significantly long carbon chains due to their high *n*_*C*_ values. This indicates the presence of either fatty acids or fatty acid esters, where a carbonyl group is present in the molecule. Higher DBE values can be explained by the presence of unsaturated fatty acids or fatty acid esters. Generally, increasing the number of oxygen atoms shifts the molecular formulae to higher DBE values and a DBE ≥ 4 can be caused by an aromatic compound. Lignin is characterized by especially high DBE values due to its aromatic structure and high oxygen numbers because of oxygen-containing functional groups, such as phenolic OH groups, methoxy groups, and ether groups (heteroatomic classes O_4_ to O_9_ in the negative ion mode). On the other hand, there are almost no compounds present in the O_2_ and O_3_ classes in the positive ion mode. As a result, GALDI(+)-MS seems not suitable for ionizing fatty acids, and the negative ion mode is preferred [33]. Basic nitrogen-containing compounds like heteroaromatics can be readily protonated, and therefore, they are better detectable in the positive ion mode [34, 35]. In the GALDI(+)-MS analyses, most nitrogen-containing compounds have DBE values greater than 10. An aromaticity of the compounds is essential to achieve those high DBEs. It is therefore assumed that condensed, heteroaromatic compounds are also a component of the EPS. In the GALDI(−)-MS analyses, only a limited number of N-containing molecular formulae were detected (see SI S5, Fig. S14).

**Fig. 5:**
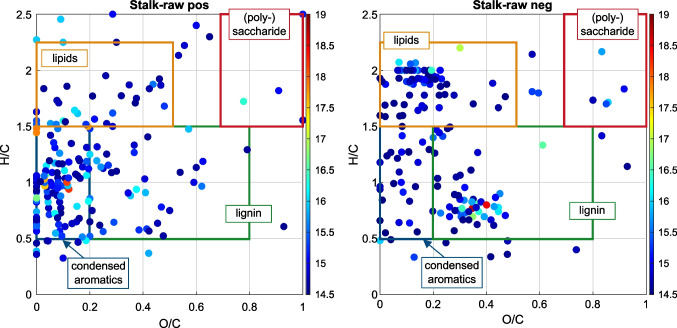
Van Krevelen plot for GALDI(−)- and GALDI(+)-FT-ICR-MS analyses of the untreated stalks (Stalk_raw), the log(intensity) of the sum formulae are presented color-coded in a range of 14.5 to 19.0, main substance classes are marked by colored rectangles

Van Krevelen plots can be used to obtain detailed information about different substance classes in the sample. For each molecular formula, the *O/C* ratio and the *H/C* ratio are calculated and plotted against each other. Each point in the van Krevelen plot represents a molecular formula. Different classes of biomolecules, such as lipids, lignin, carbohydrates, and condensed hydrocarbons, have different characteristic ratios, causing them to appear in specific areas in the van Krevelen plot. The boundaries for each compound class shown in the van Krevelen plots of the GALDI(+)- and GALDI(−)-MS results in Fig. 5 were adopted from Rivas-Ubach et al. [36] The different substance classes are represented by differently colored rectangles.

In the orange region (*O/C* = 0−0.5, *H/C* = 1.5–2.3), we can confirm the presence of lipids, such as fatty acids or fatty acid esters. Also, in the blue region (*O/C* = 0–0.2, *H/C* = 0.5–1.5), signals of condensed aromatic compounds can be identified, such as the nitrogen-containing aromatic compounds, as indicated by the *n*_*C*_-DBE plots. Signals in the green region (*O/C* = 0.2–0.8, *H/C* = 0.5–1.5) are assigned to lignin or lignin-like oligomers. As mentioned above, lignin or lignin-like oligomers could be detected especially in the negative ion mode. Using GALDI(+)-MS, lignin compounds were also detected, but the compounds appear in a different area than in the GALDI(−)-mode, indicating structural differences between the detected molecules. Carbohydrates (red rectangle, *O/C* = 0.7–1, *H/C* = 1.5–2.5) were not detected in a high abundance by either technique. These compounds are not extracted in significant amounts during the applied HR-MS sample preparation (methanol extraction).

### Fluorescence microscopy

The suggestion of lignin structures in the van Krevelen plots reported in this study was motivating to specifically search for this component using additional analytical methods. Recently, lignin was identified in the red algae *Calliarthon cheilosporioides* as a component of the secondary cell walls. This was the first finding of lignin in a non-vascular plant [37]. Lignin, which is one of the most abundant biopolymers next to cellulose and hemicellulose, is mainly found in the secondary cell wall of woods and grasses where it plays a significant role in the transportation of water throughout the plant, prevents degradation by microorganisms, and contributes to its stability and growth. Lignin is composed of aromatic phenylpropanoid moieties that are linked together to form large networks [38]. As a primary component found in many types of vascular plants, its composition varies based on the plant type. However, lignin-like molecules and monolignols are also present in non-vascular plants such as mosses (e.g., *Physcomitrella patens*) [39] or red algae [37]. Additionally, homologs of genes required for the lignin synthesis pathway were identified in green alga and diatoms [40]*.* Labeeuw et al. [40] initiated an extensive study examining the genome of various organisms to assess the presence of the enzymes necessary for lignin synthesis.

Thus, fluorescence microscopy combined with staining of the samples under study with suitable fluorescence markers was used, which offers a robust technique for the detection of lignin in biological samples. Selected cationic dyes are useful in indicating the presence of lignin [41], among which two commonly used dyes are Safranin O (SO) [42–44] and Acridine Orange (AO) [45–47]. In aqueous solutions, cationic dyes have a high affinity for acidic material, and for this reason, both dyes show a high affinity for lignin and a low affinity for pure cellulose [43]. These dyes are primarily used for selectively staining fibers. For example, SO exhibits a high relative selectivity towards lignin in comparison to cellulose and hemicellulose [43], shown for identifying lignin-rich fibers, such as those found in woods [42]. AO has also been used to stain lignin for instance in pulp fibers to gain information about the lignin distribution [45, 48]. Generally, two different concentration ranges can be used for AO that lead to different fluorescence emissions [45]. Concentrations of 1 µmol/l or less resulted in the formation of monomers, which interact with lignin, producing green fluorescence emission. Increasing the AO concentration above 1 µmol/l leads to the formation of AO-dimers, which emit red fluorescence light in combination with lignin. In addition, the presence of cellulose material in a sample leads to green fluorescence. Consequently, this method allows for distinguishing between lignified and non-lignified fibers [47].

In our study, two samples (Stalk_raw and Stalk_EDTA) were treated with a SO solution as well as two AO solutions with different concentrations (10^−6^ mol/l and 10^−2^ mol/l). In addition to the Didymo samples, a reference sample containing lignin, named Indulin, was utilized. Indulin is an unsulfonated Kraft lignin [49] that is extracted from the black liquor, a byproduct of the paper industry. The lignin is separated from the cellulose during paper pulp production by Na_2_S/NaOH and then collected in the black liquor [50].

**Fig. 6:**
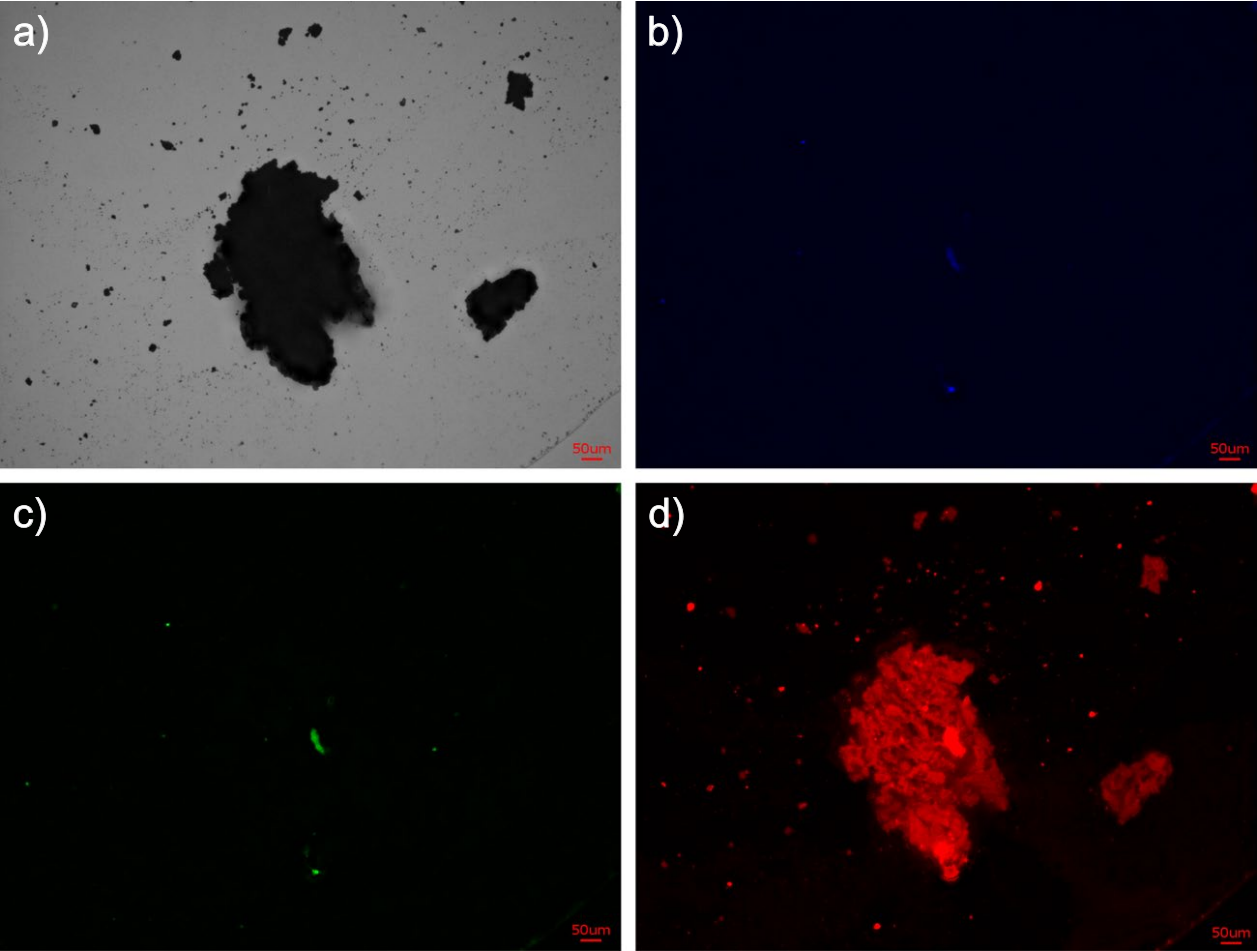
Staining of Indulin with SO (light exposure time 0.2 s); **a**) digital microscopic image, **b**) blue channel, **c**) green channel, **d**) red channel (scale bars 50 µm, image size 87 × 65.8 mm)

**Fig. 7:**
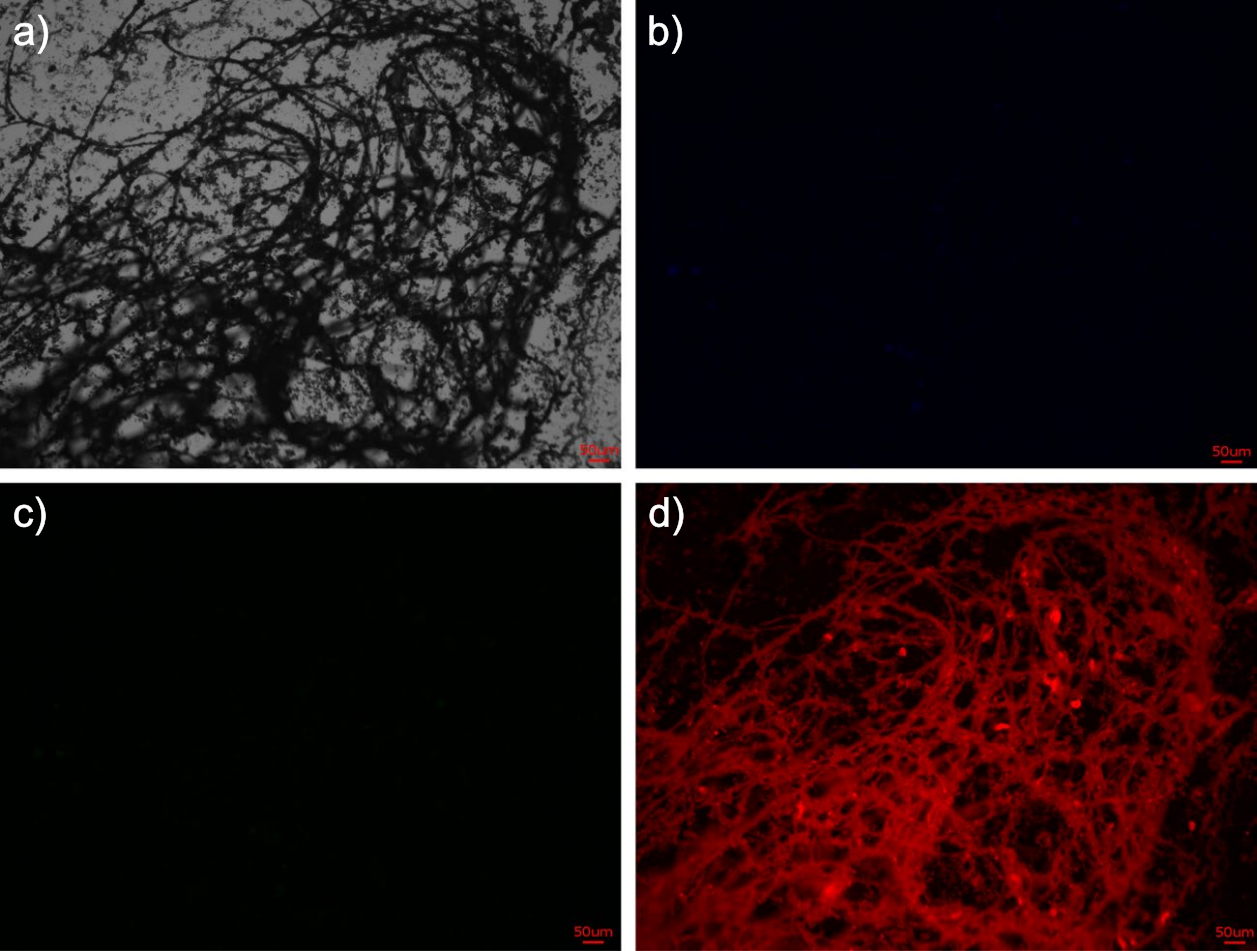
Staining of sample Stalk_raw with SO (light exposure time 0.05 s); **a**) digital microscopic image, **b**) blue channel, **c**) green channel, **d**) red channel (scale bars 50 µm, image size 87 × 65.8 mm)

All fluorescence microscopy images captured in this study are displayed in the SI S6. In the following section, only the samples stained with SO are discussed. The Indulin (Fig. 6) emits only red fluorescence due to its interaction with the SO. Only small amounts of blue and green fluorescence are visible, possibly due to some impurities in the lignin sample. Impurities cannot be avoided because the purification of natural products is challenging. Figure 7 shows the fluorescence of untreated *D. geminata* stalks (Stalk_raw) stained with SO, which shows similar results when compared to Fig. 6. There is a noticeable red fluorescence and no blue or green emission, which indicates the existence of lignin.

The digital microscopic image and the image taken from the red channel are consistent and demonstrate the fibrous structure of the stalks. The Stalk_EDTA sample (referenced in SI S6, Fig. S19) displays less distinct red coloration and there are additional impurities detected by green and blue fluorescence. This demonstrates the contamination of the samples, specifically due to the harvesting process, where complete separation of the stalks from the residue of the sampling side and the sampling process was not possible. As an example, siliceous cells still were found in both Stalk_raw and Stalk_EDTA samples (see SI S6, Fig. S19), which indicates an incomplete separation of the stalks and the siliceous cells, which are strongly attached to them.

All three samples under study showed green and blue fluorescence next to the red color when using the 10^−6^ mol/l AO solution. AO monomers cause green fluorescence. When the concentration is increased to 10^−2^ mol/l, the green fluorescence decreases (for more details, see SI S6, Fig. S20–S25).

## Conclusion

In this study, a variety of spectroscopic, spectrometric, and microscopic techniques were used to analyze organic components of the EPS of Didymo. The growth of Didymo poses a risk to the ecosystem of rivers worldwide, because its stalks can persist for a long period of time and its challenging to remove the alga and its stalks. Therefore, the comprehensive analysis of the stalk material is vital to develop potential applications and strategies for an ecofriendly pushback. In order to obtain a general overview and to showcase differences between the samples, IR spectroscopy and ^13^C-MAS-NMR spectroscopy were utilized. To further characterize the aromatic signals detected in the ^13^C-MAS-NMR spectra and to classify the analytes, HR-MS was utilized. Here, the *n*_*C*_-DBE plots and van Krevelen plots of GALDI(+)- and GALDI(−)-FT-ICR-MS data showed various molecules that can be classified as lipids, mainly fatty acids and fatty acid esters as well as aromatic compounds. The aromatic components of the stalks could be narrowed down further to the classes of condensed aromatic compounds as well as lignin and lignin-like oligomers. The fluorescence microscopy method used here additionally confirmed the presence of lignin-like molecules.

Investigations regarding the EPS of different types of marine and freshwater diatoms have been performed before, mainly with a focus on the carbohydrate composition [51]. The monosaccharide composition differs between different species. Hence, it can be assumed that they also differ in terms of other components. The EPS of Didymo consists additionally of crystalline calcite compounds and therefore appeared to be a complex biocomposite-based system made up of numerous biomolecules including lignin, and represents an example of multiphase biomineralization [4, 52]. In spite that calcium lignin composites have been already recognized (see for overview [53]), the possible role of lignin as a template for calcification with respect to the formation of nanocalcitic fibers within stalks of Didymo [4] remains to be unknown, however a very intriguing topic. The verification of lignin-like compounds is unique, and to our knowledge, this study is the first to present the identification of lignin-like compounds in an EPS of diatoms. Additionally, this study presents a new approach for the analysis of complex systems of biological origin, especially in regard to lignin compounds, and expands the possibilities for analyzing the EPS of stalk-forming diatoms. Moreover, the finding of lignin-like structures in the EPS should encourage the development of new fields of application. For instance, this finding will stimulate studies on Didymo stalks as a global source of sustainable composites, which have been recently proposed for the partial replacement of petroleum products [54, 55]. However, more research regarding the lignin content is necessary using chromatographic methods like HPLC-MS (high-performance liquid chromatography–MS) or HPLC-MS/MS to develop future applications.

In future works, extracts of different substance classes can be further characterized using liquid-state NMR and HR-MS, especially MS^n^ experiments. Additionally, electrothermal vaporization (ETV) combined with inductively coupled plasma optical emission spectroscopy (ICP-OES) will provide more information about the elemental composition and can be used to ascertain information about the diversity of elements including oxygen [56] and sulfur [57] and its different bonding forms and species. The results presented in this work provide a solid foundation for further research to explore the structural properties in the Didymo stalks and, finally, to develop measures for the removal of Didymo from rivers or limitations for its growth. Our finding of lignin within these microtubular constructs opens the key way to future studies on their large-scale potential applications, for instance as filler and food packaging material. In further works, also the analysis of samples from different geographical origins should be performed to discuss the influence of its origin on the structure of the stalks.

### Supplementary Information

Below is the link to the electronic supplementary material.

Supplementary file1 (PDF 1.80 MB)
