# Supplementary material for: Structural characterization of the extracellular stalk material of the diatom Didymosphenia geminata
Source: Anal Bioanal Chem. 2024 Jun 10;416(19):4341–52. doi: 10.1007/s00216-024-05370-1 (PMC11271372; doi:10.1007/s00216-024-05370-1)
Supplement: Supplementary file 1 — Supplementary file1 (PDF 1.80 MB) [file 216_2024_5370_MOESM1_ESM.pdf]

Supporting Information

Structural characterization of the extracellular stalk material of the diatom *Didymosphenia geminata*

published in: Analytical and Bioanalytical Chemistry

Lara Dütsch[1*], Erica Brendler[1], Jan Zuber[1*], Christine Viehweger[2], Hermann Ehrlich[3,4], Teofil Jesionowski[4], and Carla Vogt[1]

[1] *Institute of Analytical Chemistry, TU Bergakademie Freiberg, Leipziger Str. 29, 09599 Freiberg, Germany*

[2] *Institute of Geology, TU Bergakademie Freiberg, Gustav-Zeuner-Str. 12, 09599 Freiberg, Germany*

[3] *Center for Advanced Technology, Adam Mickiewicz University in Poznań, Uniwersytetu Poznańskiego 10, 61-614 Poznań, Poland*

[4] *Institute of Chemical Technology, Faculty of Chemical Technology, Poznan University of Technology, Berdychowo 4, 60-965 Poznań, Poland*

* corresponding author: Lara-Marie.Duetsch@chemie.tu-freiberg.de and Jan.Zuber@chemie.tu-freiberg.de

Contents

S1. Solid-state ^{13}C-MAS-NMR S-4

S2. Electron Paramagnetic Resonance (EPR) spectroscopy of the stalks S-8

S3. GALDI-FT-ICR-MS mass spectra of the stalks (Stalk_raw) S-11

S4. Analysis of nitrogen-, oxygen- and sulfur-containing molecular formulae S-12

S5. n_C-DBE plots of oxygen- and oxygen-/nitrogen- and oxygen-/sulfur- containing compounds S-14

S6. Fluorescence microscopy investigations S-17

List of Figures

Figure S1: ^{13}C-SP/MAS-NMR spectrum of the untreated stalks (Stalk_raw), repetition time 60 s, 4 k scans. S5

Figure S2: ^{13}C-SP/MAS-NMR spectrum of the demineralized stalks (Stalk_HCl), repetition time 1 s, 80 k scans. S5

Figure S3: ^{13}C-CP/MAS-NMR spectrum of the untreated stalks (Stalk_raw), repetition time 2 s, contact time 1 ms, 30 k scans. S6

Figure S4: ^{13}C-CP/MAS-NMR spectrum of the demineralized stalks (Stalk_HCl), repetition time 2 s, contact time 1 ms, 30 k scans. S6

Figure S5: ^{13}C-CP/MAS-NMR spectrum of the EDTA extracted, demineralized stalks (Stalk_EDTA), repetition time 2 s, contact time 1 ms, 30 k scans. S7

Figure S6: Comparison of the ^{13}C-CP/MAS-NMR spectra of the samples Stalk_HCl, Stalk_EDTA and Stalk_washed. S7

Figure S7: EPR spectrum of the sample Stalks_raw. S8

Figure S8: EPR spectrum of sample Stalks_HCl. S8

Figure S9: EPR spectrum of Stalks_EDTA. S9

Figure S10: GALDI-MS mass spectra of Stalk_raw in negative (left) and positive (right) ion mode. S11

Figure S11: Comparison of the relative abundancies of molecular formulae for the heteroatomic classes N_1, N_1O_1–N_1O_4, S_1O_2–S_1O_8 and O_2–O_{12} of the GALDI(–) and GALDI(+)-MS analyses. S12

Figure S12: Section of the GALDI(+)-mass spectrum, m/z of 490 to 550, peaks assigned to N_1O_4 class colored in red. S13

Figure S13: Section of the GALDI(–)-mass spectrum, m/z of 200 to 600, peaks assigned to different oxygen-containing groups colored in red (O_3), green (O_4), violet (O_9) and light blue (O_{12}). S13

Figure S14: n_C-DBE plots for the heteroatomic classes N_1, N_1O_1–N_1O_4, S_1O_2–S_1O_8 and O_2–O_{12}, from GALDI(–)-MS analysis, the log(intensity) of the DBE values are presented color-coded in a range of 14.5–18. .... S14

Figure S15: n_C-DBE plots for the heteroatomic classes N_1, N_1O_1–N_1O_4, S_1O_2–S_1O_8 and O_2–O_{12}, from GALDI(+)-MS analysis, the log(intensity) of the DBE values are presented color-coded in a range of 14.5–18. .... S15

Figure S16: Structure of Acridine Orange (left) and Safranin O (right). S17

Figure S17: Staining of Indulin with SO (light exposure time 200 ms); a) digital microscopic image, b) blue channel, c) green channel, d) red channel (scale bar 50 µm, image size 146 x 110 mm). S18

Figure S18: Staining of Stalk_raw with SO (light exposure time 50 ms); a) digital microscopic image, b) blue channel, c) green channel, d) red channel (scale bar 50 µm, image size 146 x 110 mm). S18

Figure S19: Staining of Stalk_EDTA with SO (light exposure time 30 ms); a) digital microscopic image, b) blue channel, c) green channel, d) red channel (scale bar 50 µm, image size 146 x 110 mm). S19

Figure S20: Staining of Indulin with AO (low) (light exposure time 200 ms); a) digital microscopic image, b) blue channel, c) green channel, d) red channel (scale bar 50 µm, image size 146 x 110 mm). S19

Figure S21: Staining of Stalk_raw with AO (low) (light exposure time 200 ms); a) digital microscopic image, b) blue channel, c) green channel, d) red channel (scale bar 50 µm, image size 146 x 110 mm). S20

Figure S22: Staining of Stalk_EDTA with AO (low) (light exposure time 250 ms); a) digital microscopic image, b) blue channel, c) green channel, d) red channel (scale bar 50 µm, image size 146 x 110 mm). S20

Figure S23: Staining of Indulin with AO (high) (light exposure time 250 ms); a) digital microscopic image, b) blue channel, c) green channel, d) red channel (scale bar 50 µm, image size 146 x 110 mm). S21

Figure S24: Staining of Stalk_raw with AO (high) (light exposure time 4.0 ms); a) digital microscopic image, b) blue channel, c) green channel, d) red channel (scale bar 50 µm, image size 146 x 110 mm)........................... S21

Figure S25: Staining of Stalk_EDTA with AO (high) (light exposure time 200 ms); a) digital microscopic image, b) blue channel, c) green channel, d) red channel (scale bar 50 µm, image size 146 x 110 mm). S22

List of Tables

Table S1: Assignments of the important regions in the solid-state ^{13}C-MAS-NMR spectra. S-4

Table S2: Results of the ICP-OES analysis of the sample Stalks_EDTA after a microwave digestion using HF, three-fold determination, elements with a content lower than the limit of quantification (LOQ) are colored red... ....S-9

Table S3: Masses of all samples for each experiment..S-17

S1. Solid-state ^{13}C-MAS-NMR

NMR spectroscopic investigations were performed of the stalks of *Didymosphenia geminata*. Of the untreated stalks and the demineralized sample, single pulse excitation (SP) spectra were recorded using the *hpdec* pulse program (Figure S1 and Figure S2). The crystalline calcite has a long relaxation time and therefore the repetition time was set to 60 s to achieve a complete relaxation of all ^{13}C nuclei in the calcite. However, the long recycle delays lead to long experimental times and thus, only 4 k scans could be recorded in a reasonable time (approximately 3 days). By treating the stalks with a 6 M hydrochloric acid solution, the calcite was dissolved and could be removed from the samples. This way and due to the presence of paramagnetic ions in the material, the repetition time could be reduced to 1 s and more scans could be recorded. In addition to SP/MAS-NMR spectra, CP/MAS-NMR spectra were recorded giving a better SNR. However, the calcite signal cannot be detected by this method. The different regions in the spectra can be assigned to typical structural groups. The regions used here for the analysis of all spectra are summarized in the following Table S1. All spectra were recorded at a 400 MHz WB spectrometer in 4 mm ZrO_2 rotors at 10 kHz rotation frequency and with tppm15 decoupling. The results are presented in the Figure S1 to Figure S5.

Table S1: Assignments of the important regions in the solid-state ^{13}C-MAS-NMR spectra.

**chemical shift in ppm**	**assignment**
190–165	carbonyls (mainly esters and carboxylic acids)
140–120	aromatic units
105–95	anomeric carbon of carbohydrates (C_1)
80–60	C_2 to C_6 of carbohydrates
<40	aliphatic components

In the ^{13}C-CP/MAS-NMR spectrum of the sample Stalk_EDTA, prominent signals assigned to EDTA were observed, as stated in the main part of this paper. Therefore, the removal of EDTA was attempted by washing the stalks with distilled water three times and removing the aqueous phase by centrifugation. Subsequently, the sample (Stalks_washed) was dried and a CP/MAS-NMR spectrum analogous to the sample Stalks_EDTA was obtained. By comparing the spectra of Stalks_HCl (red), Stalks_EDTA (green) and Stalks_washed (blue), it was established that the EDTA removal was incomplete (see Figure S6). The signal at 72 ppm, assigned to the carbohydrate matrix and unaffected by the extraction experiment, was used for normalizing the spectra. Signal intensity comparison of the carbonyl region at 173 ppm indicates the removal of more than 50 % of the remaining EDTA. The removal of paramagnetic ions resulted in an increase of the intensity of signals in the aromatic (140 ppm to 120 ppm) and aliphatic (40 ppm to 10 ppm) regions. Signals of lignin and condensed aromatic compounds are expected in the aromatic region. Lipids such as fatty acids and fatty acid esters are for example responsible for signals in the aliphatic region, as determined by the MS analyses (see section S5). The aforementioned compound classes can complex paramagnetic ions like iron ions, leading to a worse magnetization transfer due to fast relaxation in the CP/MAS-NMR experiment and consequent signal intensity reduction. Removing paramagnetic compounds may result in increased signal intensity. However, the use of EDTA as a complexing agent presents new issues, as previously mentioned, due to the inability to be fully removed from the sample, resulting in signal overlapping with the sample's signals.

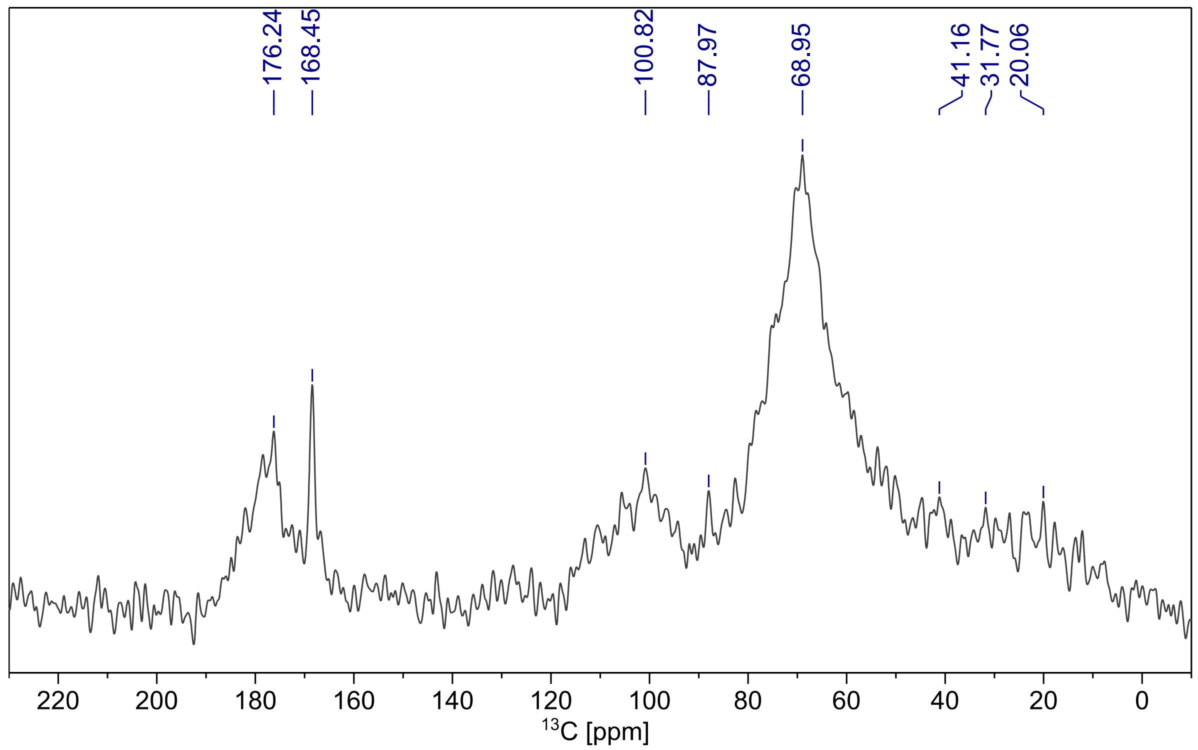

Figure S1: ^{13}C-SP/MAS-NMR spectrum of the untreated stalks (Stalk_raw), repetition time 60 s, 4 k scans.

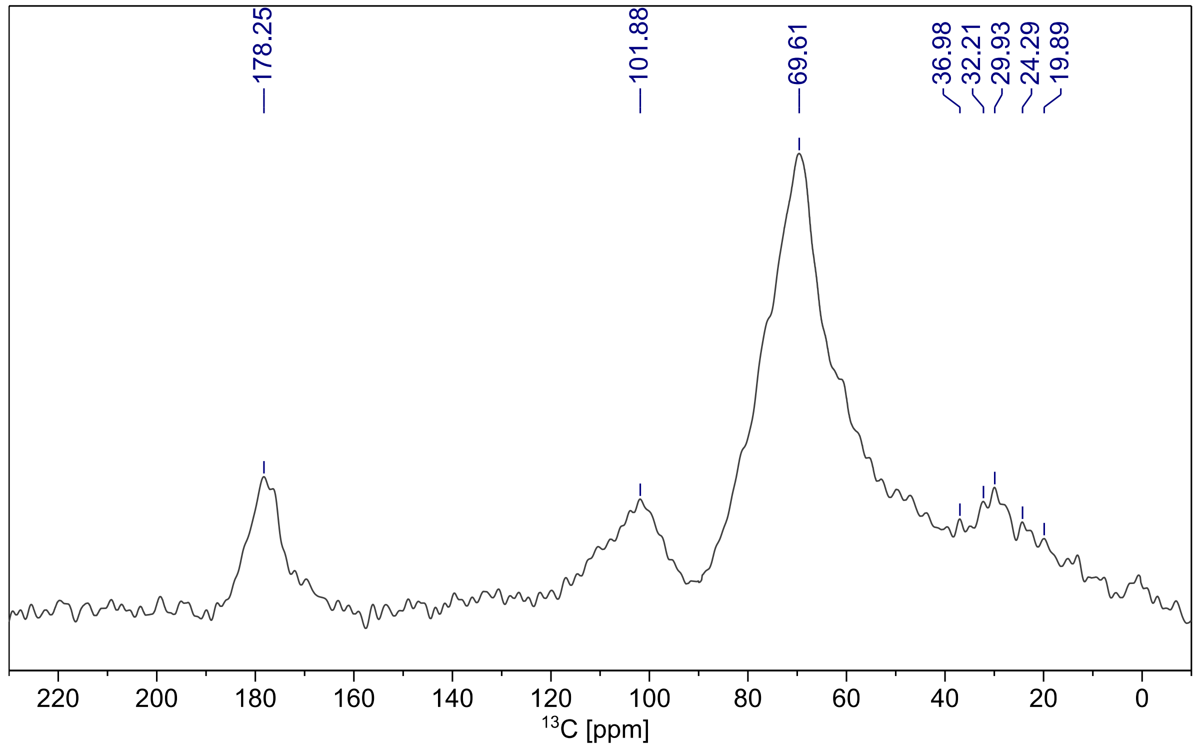

Figure S2: ^{13}C-SP/MAS-NMR spectrum of the demineralized stalks (Stalk_HCl), repetition time 1 s, 80 k scans.

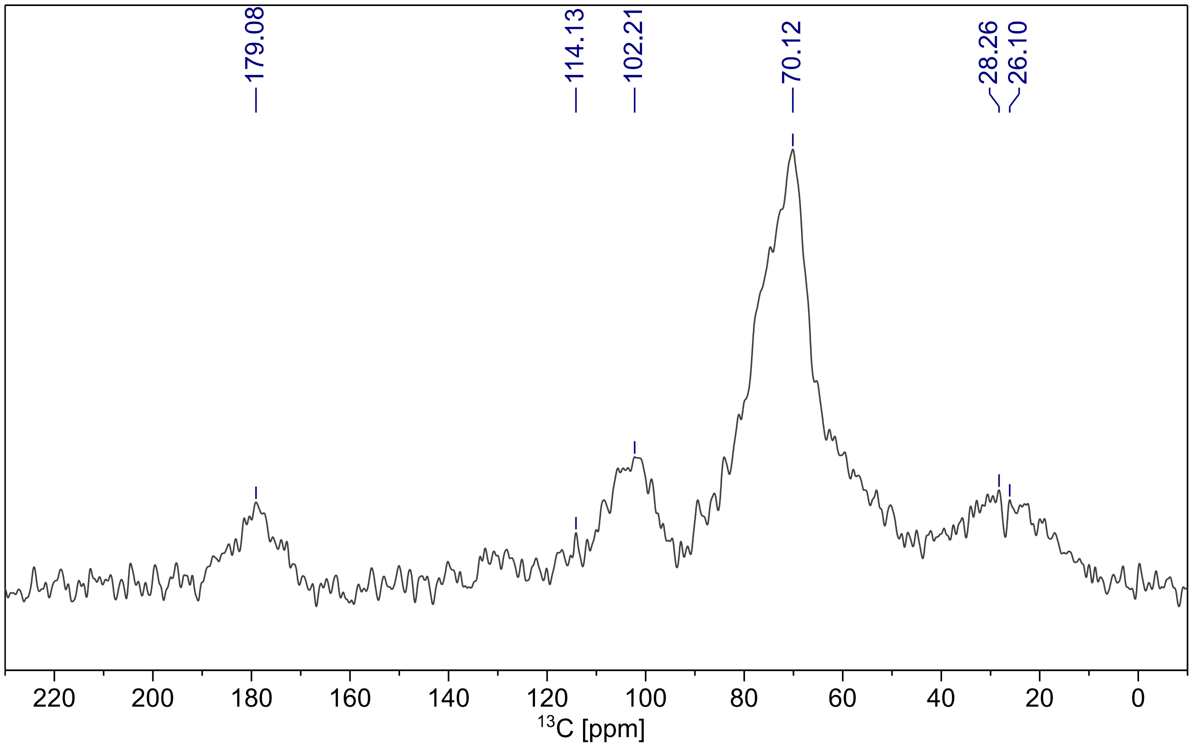

Figure S3: ^{13}C-CP/MAS-NMR spectrum of the untreated stalks (Stalk_raw), repetition time 2 s, contact time 1 ms, 30 k scans.

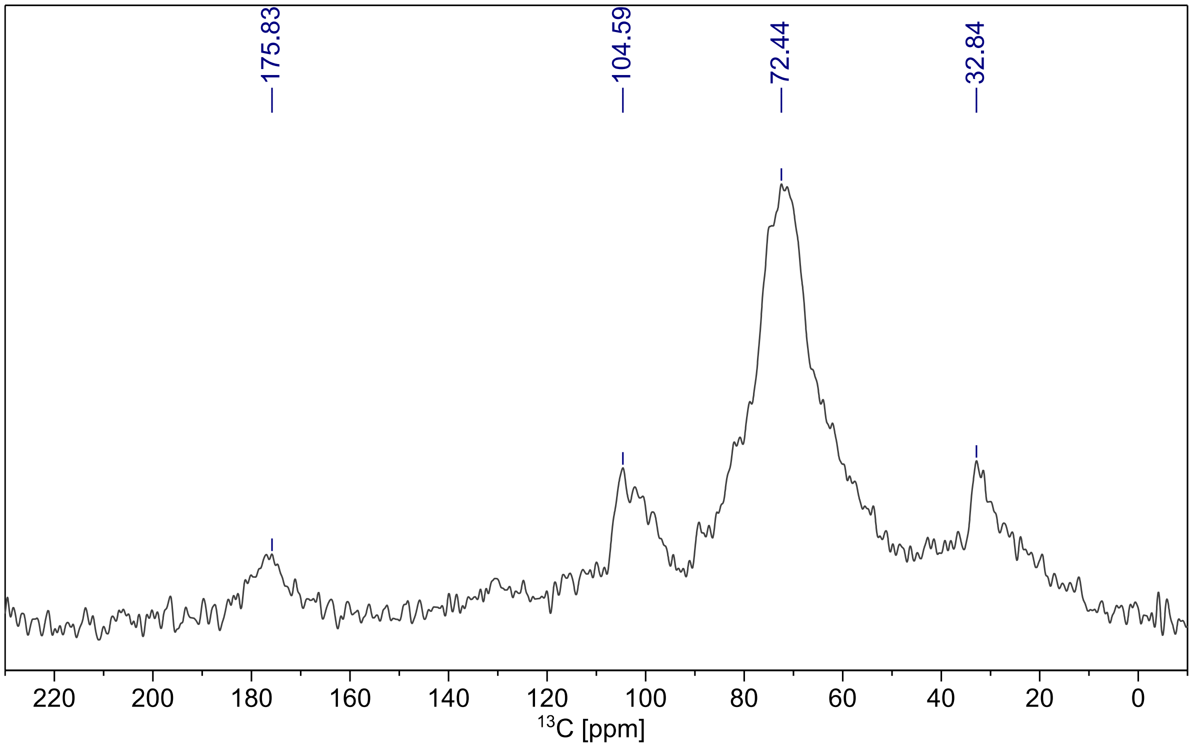

Figure S4: ^{13}C-CP/MAS-NMR spectrum of the demineralized stalks (Stalk_HCl), repetition time 2 s, contact time 1 ms, 30 k scans.

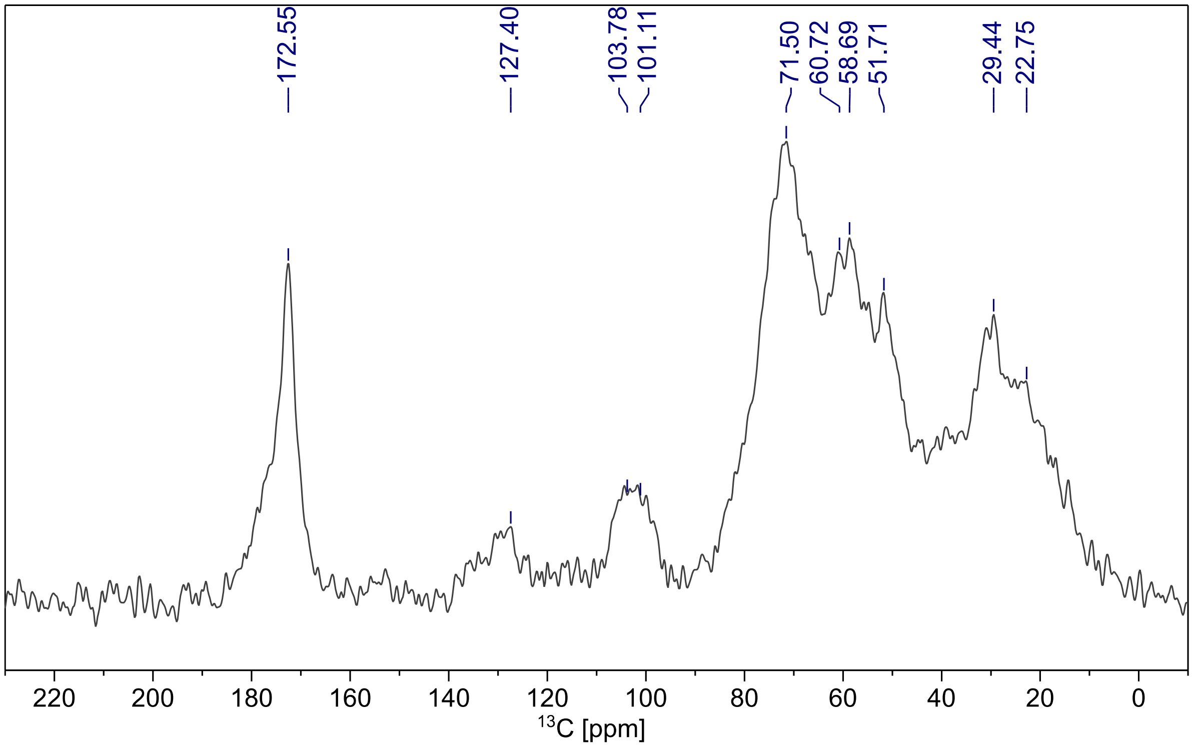

Figure S5: ^{13}C-CP/MAS-NMR spectrum of the EDTA extracted, demineralized stalks (Stalk_EDTA), repetition time 2 s, contact time 1 ms, 30 k scans.

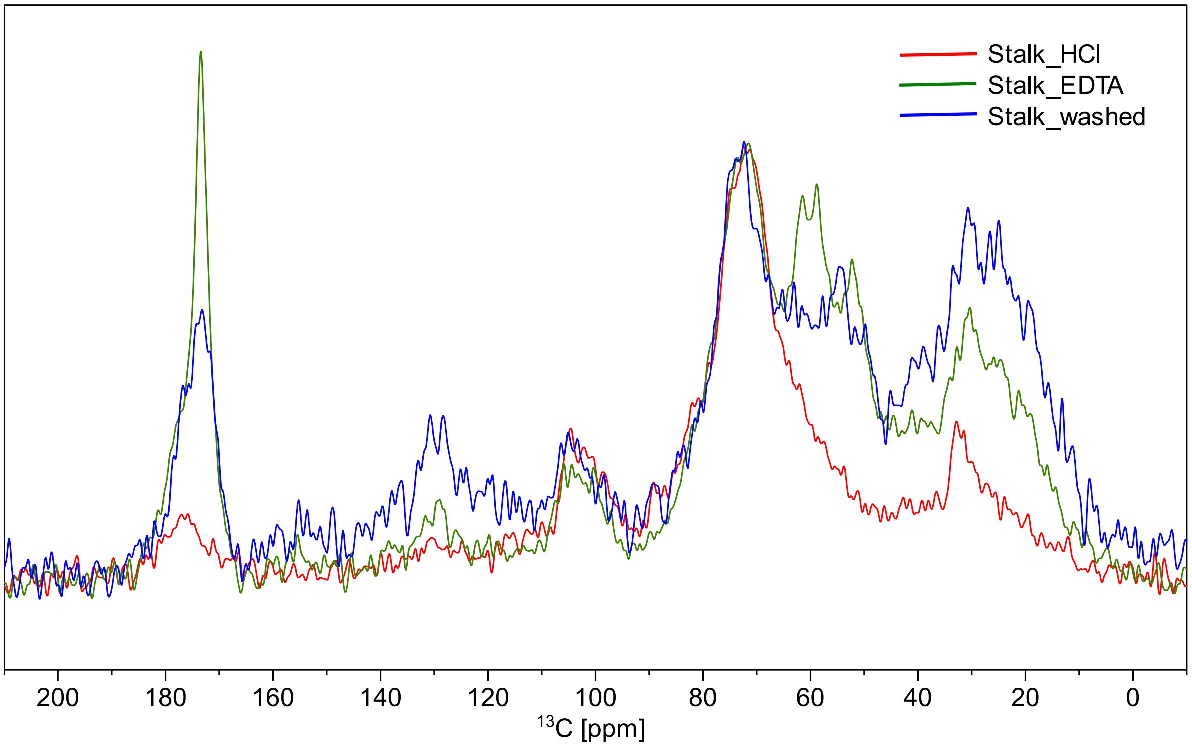

Figure S6: Comparison of the ^{13}C-CP/MAS-NMR spectra of the samples Stalk_HCl, Stalk_EDTA and Stalk_washed.

S2. Electron Paramagnetic Resonance (EPR) spectroscopy of the stalks

The solid-state NMR spectra showed broad signals indicating the presence of paramagnetic components. The carboxylic acids and the sulfoxide groups are able to complex transition metal ions such as iron. To gain further insight into the paramagnetic ion content of the samples, EPR spectra were recorded. A *MS 5000* benchtop ESR spectrometer from *Freiberg Instruments* was used. The spectra were recorded and further processed using *Freiberg Instruments*' software *ESR Studio*. A few mg of the sample were sealed in a sample tube and inserted into the spectrometer. A constant microwave field with a frequency of 9.43 GHz was applied and the magnetic field was varied between 100 to 500 mT. The microwave power was set to 10 mW and the modulation frequency was 100 kHz and the modulation field was set to 0.5 mT. The experiment time was 120 s. The EPR spectrum of all three samples are shown in Figure S7 to Figure S9. A baseline correction was performed using the tool in the software *ESR Studio*.

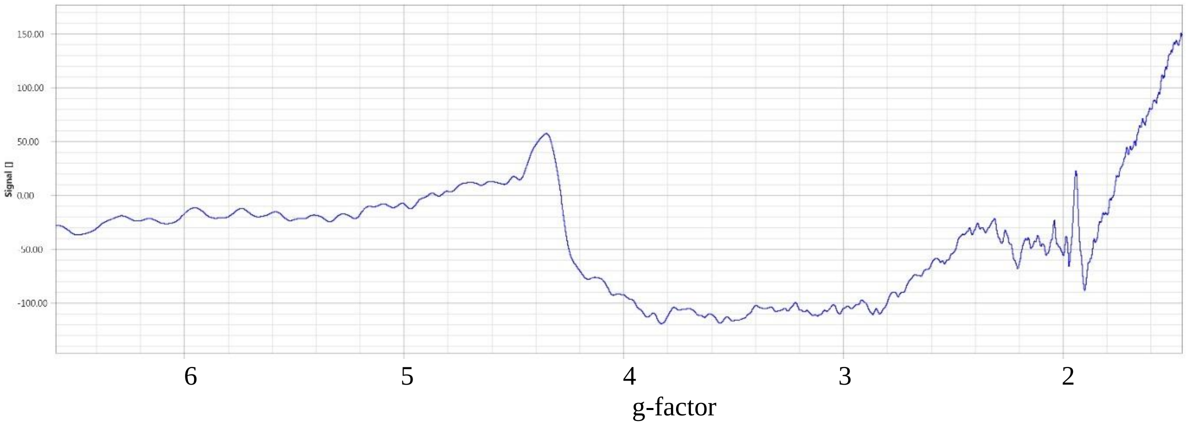

Figure S7: EPR spectrum of the sample Stalks_raw.

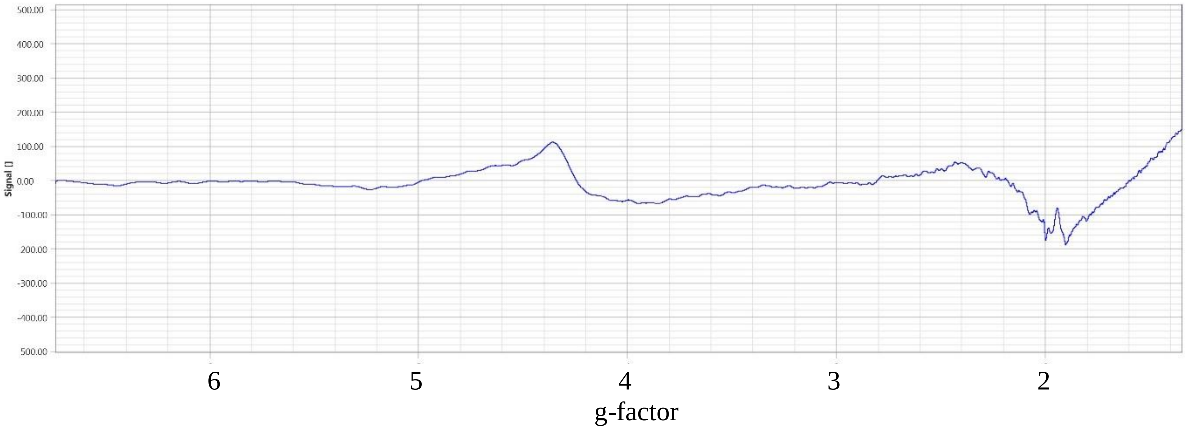

Figure S8: EPR spectrum of sample Stalks_HCl.

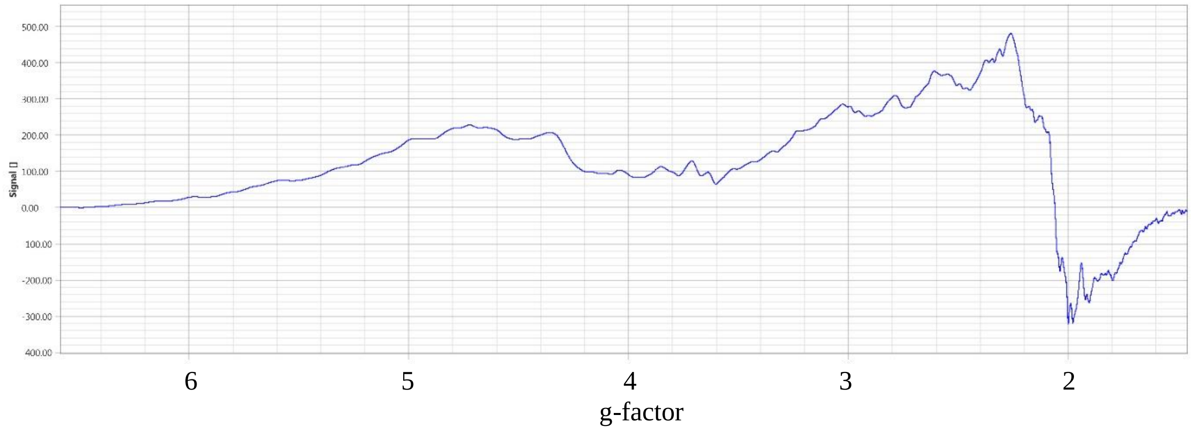

Figure S9: EPR spectrum of Stalks_EDTA.

In all three samples are signals at a g-factor of 2.0 and 4.3 approximately. The g-factor of a free electron is 2.00232[1]. Generally, the signal at a g-factor of 2.0 cannot be properly assigned, because a variety of compounds and ions generate a signal in this region, e.g. organic radicals or a wide range of transition metal ions. However, it might originate from octahedral coordinated iron(III). The signal at 4.3 can be assigned to iron(III) with tetrahedral coordination.[2] To further confirm the complexing of iron ions, ICP-OES analyses were performed after a microwave digestion using HF of the samples. The results of the sample extracted with EDTA (Stalks_EDTA) are presented in the following Table S2. The sample contained the lowest content of transition metal ions due to the extraction with EDTA.

Table S2: Results of the ICP-OES analysis of the sample Stalks_EDTA after a microwave digestion using HF, three-fold determination, elements with a content lower than the limit of quantification (LOQ) are colored red.

	elemental content [ppm]	s [ppm]	RSD [%]
**Al**	54162,35	700,45	1,3
**As**	< LOQ		
**Ba**	121,10	1,50	1,2
**Ca**	197,91	1,13	0,6
**Cd**	< LOQ		
**Co**	< LOQ		
**Cr**	< LOQ		
**Cu**	< LOQ		
**Fe**	27617,95	495,48	1,8
**K**	12423,04	26,21	0,2
**Li**	462,11	16,75	3,6
**Mg**	9813,13	76,19	0,8
**Mn**	< LOQ		
**Na**	24262,31	551,26	2,3
**Ni**	< LOQ		
**P**	217,40	2,53	1,2
**Pb**	< LOQ		
**S**	1538,36	15,14	1,0
**Si**	129855,12	2811,28	2,2
**Sr**	< LOQ		
**Ti**	1189,09	7,92	0,7
**Zn**	< LOQ		

As the results in Table S2 show, the content of most transition metal ions is below the LOQ and hence, a quantification of these elements is not possible. The content of silicon, titanium and aluminum in the sample probably originates from both the cell bodies (in regard to the silicon) as well as ground rock that is still present in the sample. After the extraction of the sample using EDTA, there is still a high iron content. Thus, we conclude that iron is complexed at the carboxylic acid groups as well as S-containing groups like sulfonic acid or sulfate ester groups. Therefore, the signal detected in the EPR spectra of all three samples presented here are assigned to tetrahedral iron(III) ions.

S3. GALDI-FT-ICR-MS mass spectra of the stalks (Stalk_raw)

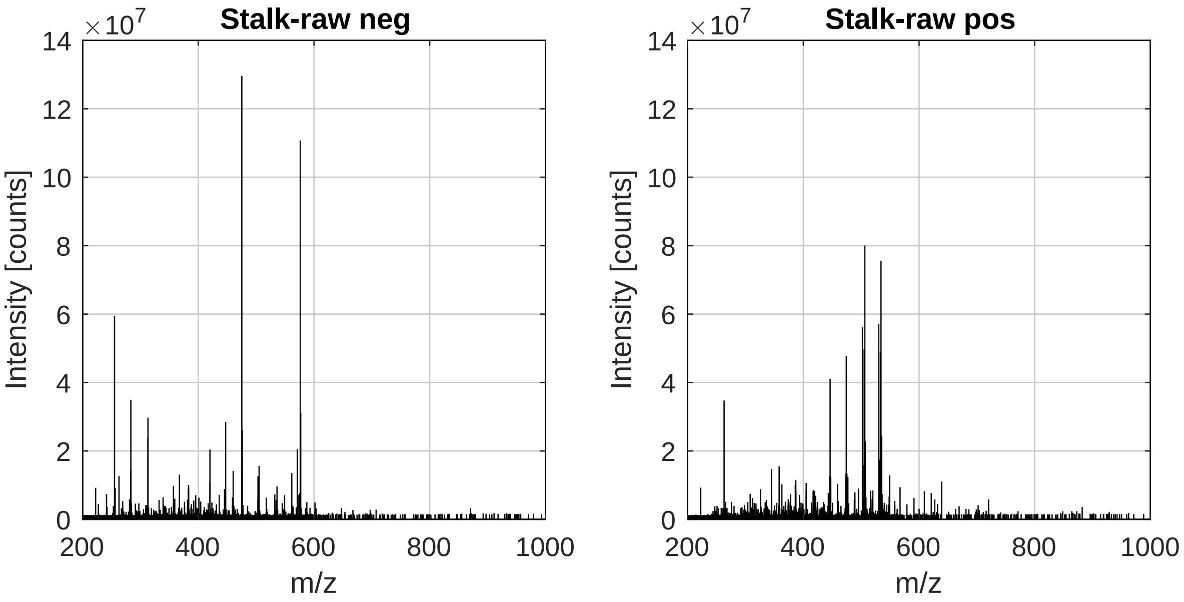

Figure S10: GALDI-MS mass spectra of Stalk_raw in negative (left) and positive (right) ion mode.

In Figure S10, GALDI(–) and GALDI(+) mass spectra are shown of the untreated stalks sample. After a blank correction, 2397 peaks were observable in the GALDI(–)-FT-ICR-MS analyses and 2606 peaks in the GALDI(+)-FT-ICR-MS analyses.

S4. Analysis of nitrogen-, oxygen- and sulfur-containing molecular formulae

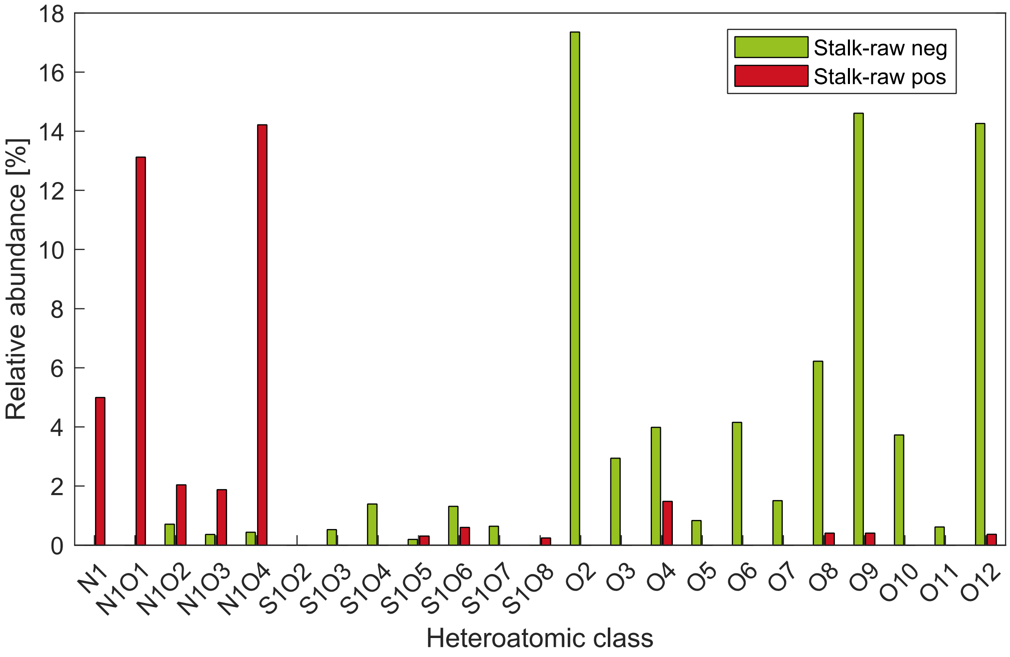

Figure S11: Comparison of the relative abundancies of molecular formulae for the heteroatomic classes N_1, N_1O_1–N_1O_4, S_1O_2–S_1O_8 and O_2–O_{12} of the GALDI(–) and GALDI(+)-MS analyses.

In Figure 3 in the main part of this paper, the total count of molecular formulae that can be assigned to each heteroatomic class is presented. Furthermore, for each heteroatomic class, the relative abundance of molecular formulae can be shown alongside to the absolute count (see Figure S11). Here, the intensity of a peak assigned to a specific molecular formula influences the plot. If an ion has a high intensity in the spectrum, it results in a high relative abundance, even if this molecular formula is the sole molecular formula detected in this class. This can be illustrated by referencing Figure S12 and Figure S13. In the positive ion mode, the heteroatom class N_1O_4 has only five molecular formulae assigned to it, making it the class with the fewest nitrogen-containing compounds. However, the relative abundance of the N_1O_4 class is the highest, surpassing 14%. This implies that at least one of the five compounds has a high signal intensity in the mass spectrum. An in-house *Matlab* script was used to visualize the overall data set and peaks of the N_1O_4 class. The mass spectrum shows two signals with m/z values of 506.232445 Da ($C_{33}H_{31}N_1O_4$) and 534.263685 Da ($C_{35}H_{35}N_1O_4$), both of which belong to compounds in the N_1O_4 class and are the most intense signals in the GALDI(+) mass spectrum (Figure S12).

A reversed trend is visible in the heteroatomic classes O_3 (red) and O_4 (green) in the negative ion mode (Figure S13). Here, both classes show a high number of molecular formulae in each class, however, the relative intensity in the mass spectrum is low in comparison to other oxygen-containing classes like O_9 (violet) and O_{12} (light blue).

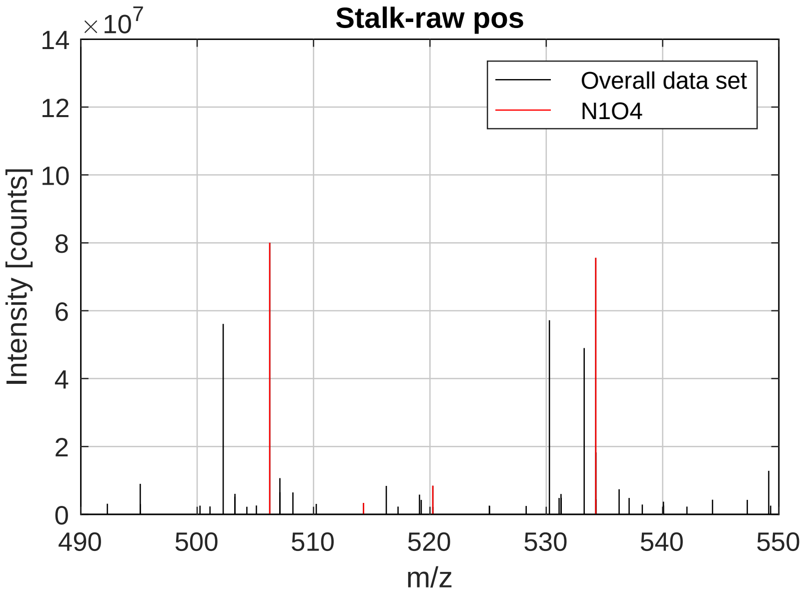

Figure S12: Section of the GALDI(+)-mass spectrum, m/z of 490 to 550, peaks assigned to N_1O_4 class colored in red.

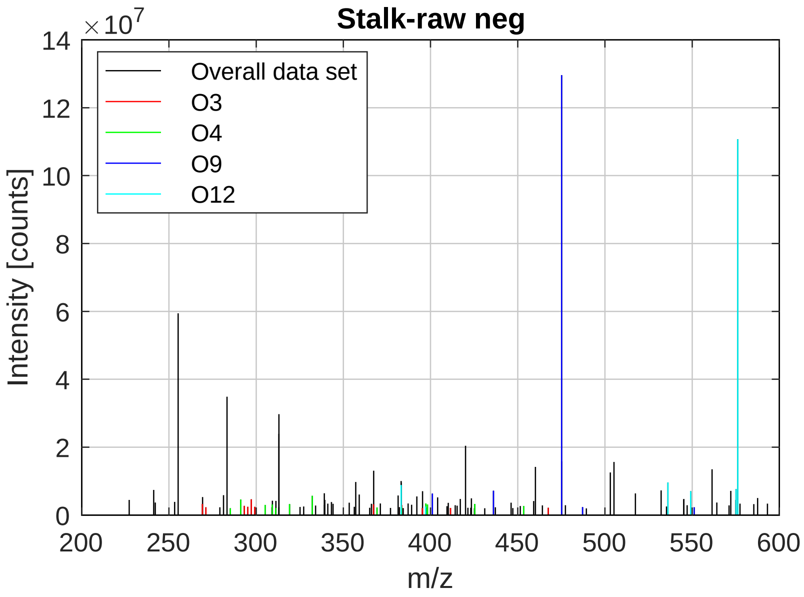

Figure S13: Section of the GALDI(–)-mass spectrum, m/z of 200 to 600, peaks assigned to different oxygen-containing groups colored in red (O_3), green (O_4), violet (O_9) and light blue (O_{12}).

S5. n_C-DBE plots of oxygen- and oxygen-/nitrogen- and oxygen-/sulfur- containing compounds

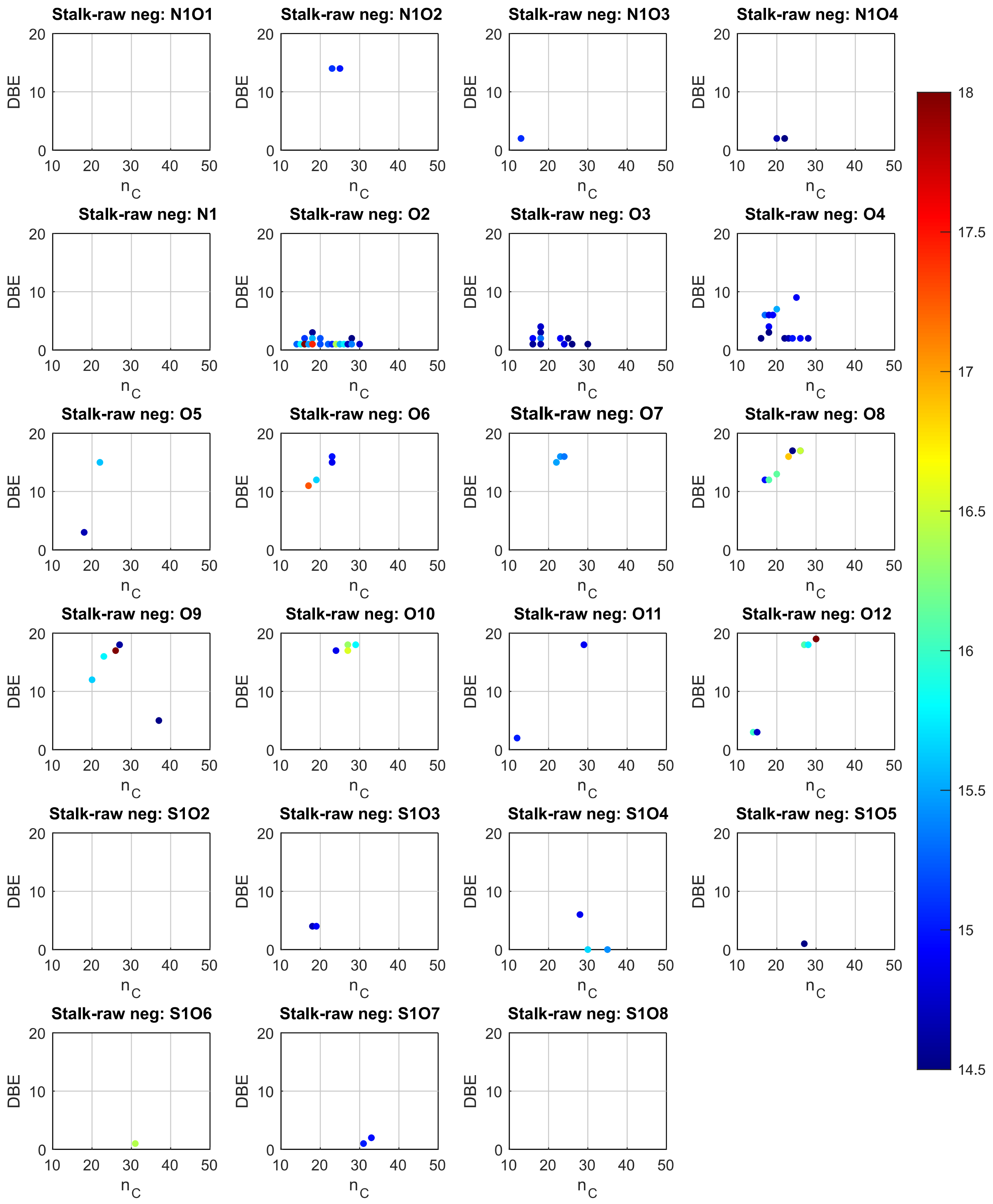

Figure S14: n_C-DBE plots for the heteroatomic classes N_1, N_1O_1–N_1O_4, S_1O_2–S_1O_8 and O_2–O_{12}, from GALDI(−)-MS analysis, the log(intensity) of the DBE values are presented color-coded in a range of 14.5–18.

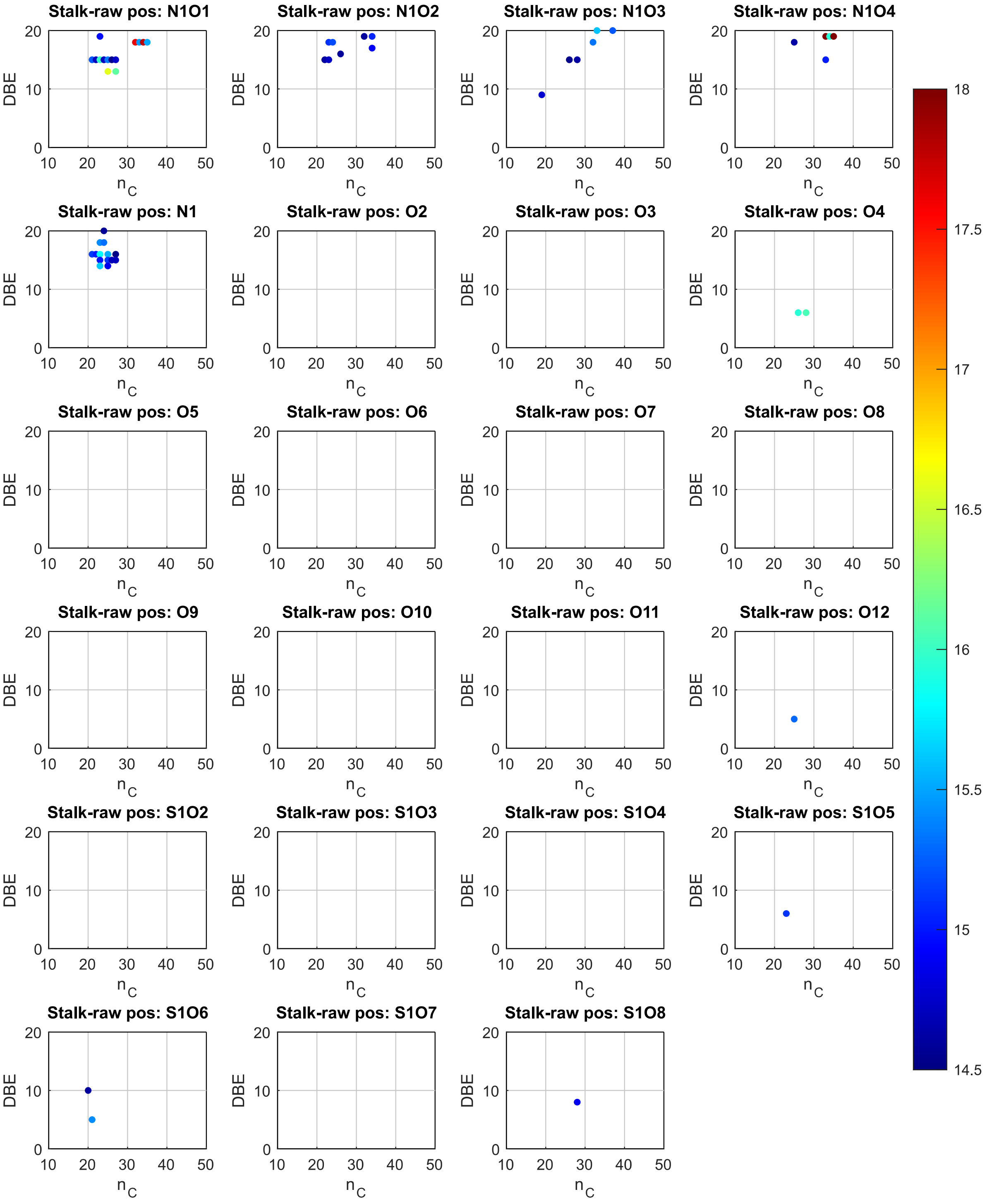

Figure S15: n_C-DBE plots for the heteroatomic classes N_1, N_1O_1–N_1O_4, S_1O_2–S_1O_8 and O_2–O_{12}, from GALDI(+)-MS analysis, the log(intensity) of the DBE values are presented color-coded in a range of 14.5–18.

The figures of the n_C-DBE plots for each heteroatomic class, which were analyzed in S4, are displayed in Figure S14 and Figure S15. The DBE was calculated using the equation (2) for a compound with a following composition of $C_cH_hO_oN_nX_x$.

$$DBE = \frac{2\,c - h + n - x + 2}{2} \qquad (2)$$

The data shows significant differences between the negative and positive ion modes. Within the negative ion mode, especially compounds within heteroatomic classes that only contain oxygen as heteroatoms are detected. As

previously stated in this paper's main section, molecular formulae with an oxygen number of two to three can be assigned to fatty acids and fatty acid esters. In the heteroatomic classes O_5 to O_{12} in Fig. S12 ions with a DBE greater 10 were detected. In combination with the high oxygen-content, these structures are assigned to lignin-like molecules. A single monolignol has at least a DBE of 5 (aromatic ring structure and a single double bond in the aliphatic propanoid-residue). Compounds in the mentioned oxygen classes are better ionized in the negative ion mode because lignin-like molecules are easily deprotonated. The oxygen-containing classes O_6 to O_{10} included the highest number of lignin-like structures and the sum formula with a DBE greater 10 and a carbon number greater 18 indicates oligomers containing two to three monolignol-units. Compounds with higher oxygen numbers and n_C values, but lower DBE values, can also be found in the classes O_{11} and O_{12}. For instance, a compound with an m/z of 341.108826 Da and a calculated molecular formula of $C_{12}H_{22}O_{11}$ is found in O_{11} and is characteristic of a disaccharide. Similar compounds are present in the O_{12} oxygen class. The peak at m/z 383.119477 Da can be assigned to $C_{14}H_{24}O_{12}$ which is probably an acetylated disaccharide. Similarly, the peak at 397.135042 Da ($C_{15}H_{26}O_{12}$) can be assigned to a modified disaccharide. However, distinguishing between different monosaccharides or disaccharides with identical molecular formulae is not possible via mass spectrometry because the differences between each stereoisomer is the configuration of the hydroxy groups. Here, a fragmentation of different monosaccharide or disaccharide references is necessary to obtain their characteristic fragmentation pattern.

Using the positive ion mode, almost no molecular formulae containing only oxygen as a heteroatom could be detected. However, as mentioned in the main part of the paper, many of the detected compounds contain nitrogen and due to the high DBE, heteroaromatic compounds are most likely present in the sample. Molecular formulae containing sulfur are ionized and detected using both GALDI(+)- and GALDI(−)-MS.

S6. Fluorescence microscopy investigations

Different experiments were performed to investigate the fluorescence emission of the stalks after staining. Here, the two samples Stalk_raw and Stalk_EDTA as well as the lignin reference Indulin were stained with Safranin O (SO) and Acridine Orange (AO). Two differently concentrated AO solutions were used (0.01 M and 10^{-6} M), because depending on the concentration of AO, it is forming either monomers or dimers (see main part of this paper). The structural formula of each dye is shown in Figure S16. The sample weights used for the experiments are summarized in Table S3.

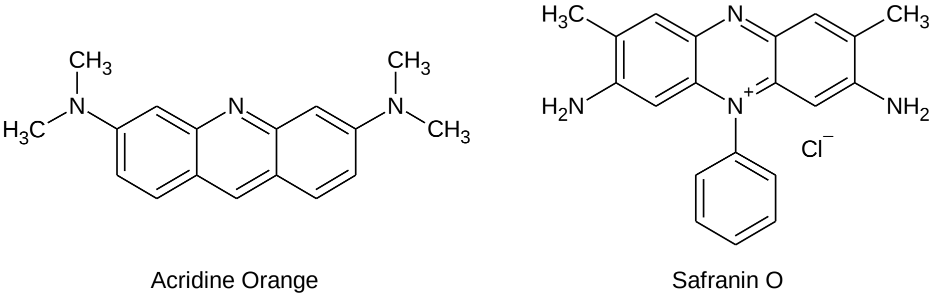

Figure S16: Structure of Acridine Orange (left) and Safranin O (right).

Table S3: Masses of all samples for each experiment.

	**SO**	**AO (high)**	**AO (low)**
**Stalk_raw**	2.9 mg	3.0 mg	2.7 mg
**Stalk_EDTA**	5.8 mg	5.3 mg	5.5 mg
**Indulin**	5.8 mg	6.8 mg	5.4 mg

SO is selective for staining lignin and indicates a red fluorescence and no green or blue emission. In the following Figure S17 to Figure S19, the results of the samples stained with SO are summarized. Indulin and Stalk_raw show similar results that indicate the presence for lignin in the stalks. The demineralized and extracted sample Stalk_EDTA lost its fibrous structure. Additionally, in the digital microscopic image in Figure S19 a) there is a fiber that does not belong to the stalk because of its different surface structure. The fiber has a prominent blue fluorescence that distinguishes it from the sample.

Two different concentrations of AO were used in the next experiment. Li and Reeve[3] noted using concentrations below 10^{-6} mol/l lead to the formation of monomers of AO only resulting in green emission when lignin is present in a sample. Increasing the concentration of AO results in the formation of AO dimers that emit red fluorescence. When cellulose is present instead of lignin, isolated AO can be adsorbed resulting in green emission even at higher AO concentrations.[4]

Looking at the results of the 10^{-6} M AO staining solution, green fluorescence is visible in both the Indulin reference and the untreated stalks (Stalk_raw). This confirms the hypothesis mentioned above that lower concentrations lead to the adsorption of monomers and a green fluorescence. Probably, dimers are also formed, leading to the red coloring that is visible in Figure S20 d), Figure S21 d) and Figure S22 d). When the concentration is increased to 10^{-2} M, there is no green fluorescence, so only dimers are formed, which interact with the lignin (Figure S23 to Figure S25).

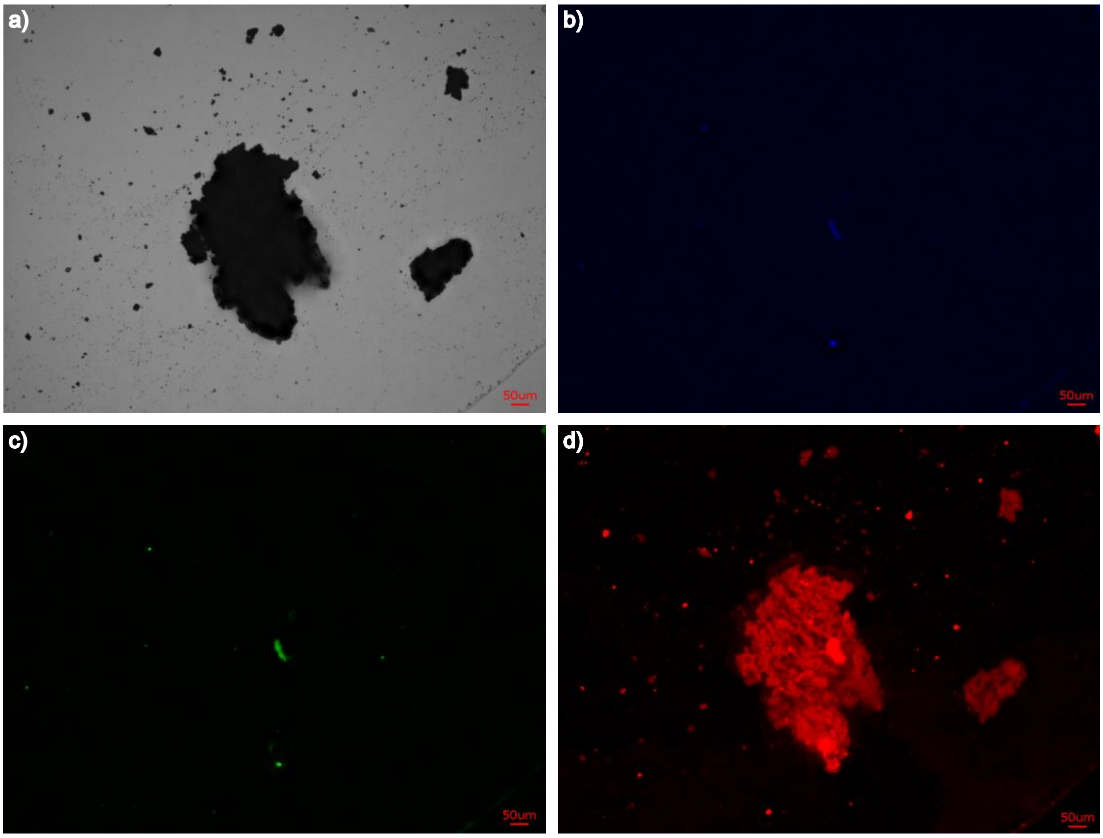

Figure S17: Staining of Indulin with SO (light exposure time 200 ms); a) digital microscopic image, b) blue channel, c) green channel, d) red channel (scale bar 50 µm, image size 146 x 110 mm).

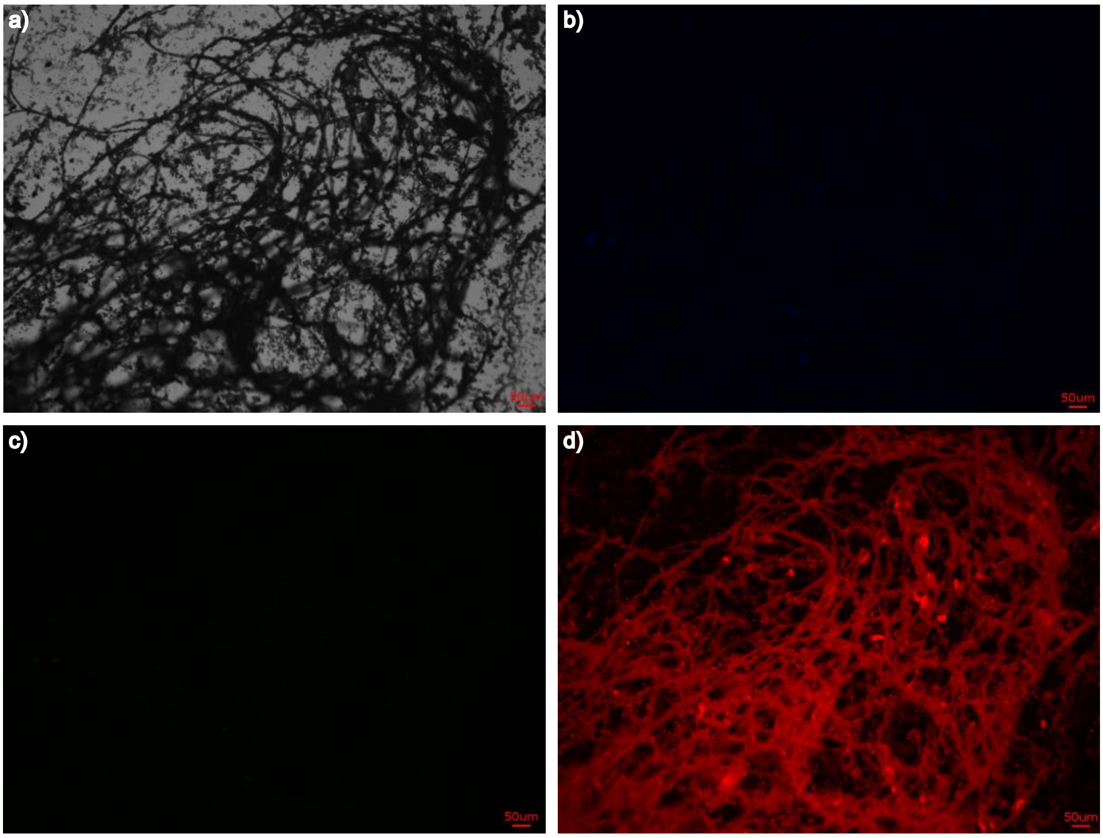

Figure S18: Staining of Stalk_raw with SO (light exposure time 50 ms); a) digital microscopic image, b) blue channel, c) green channel, d) red channel (scale bar 50 µm, image size 146 x 110 mm).

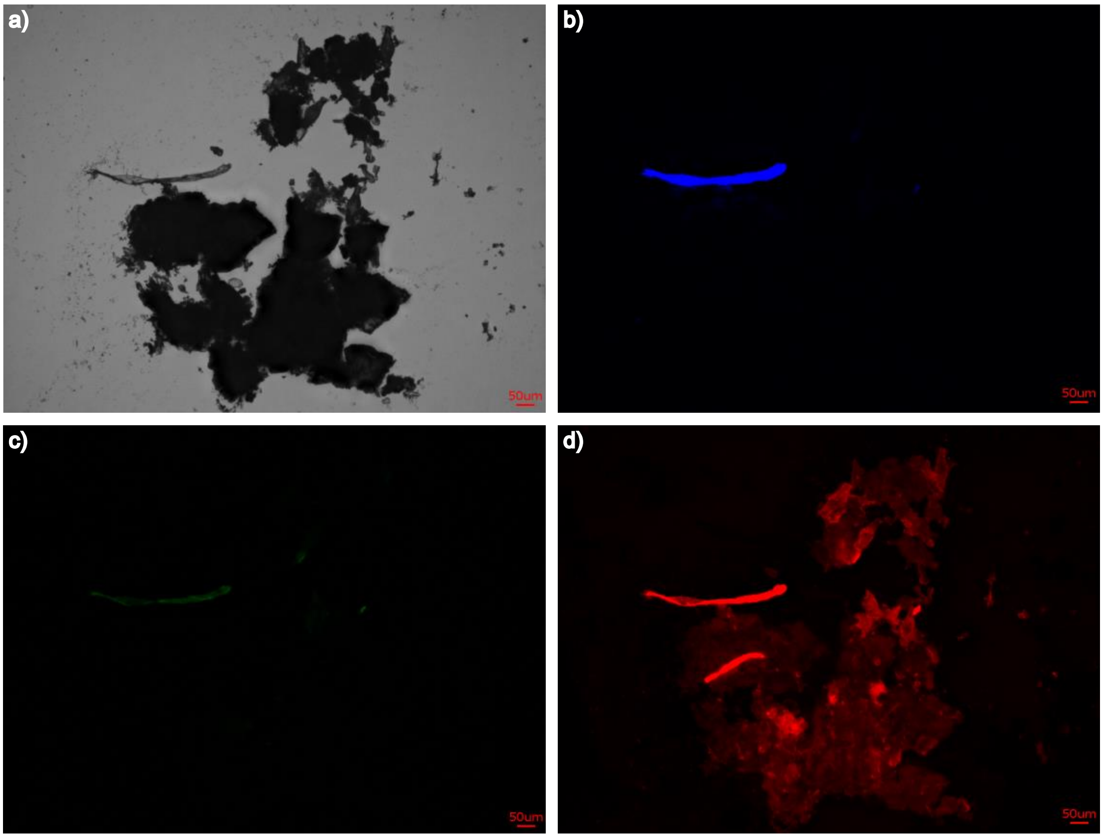

Figure S19: Staining of Stalk_EDTA with SO (light exposure time 30 ms); a) digital microscopic image, b) blue channel, c) green channel, d) red channel (scale bar 50 µm, image size 146 x 110 mm).

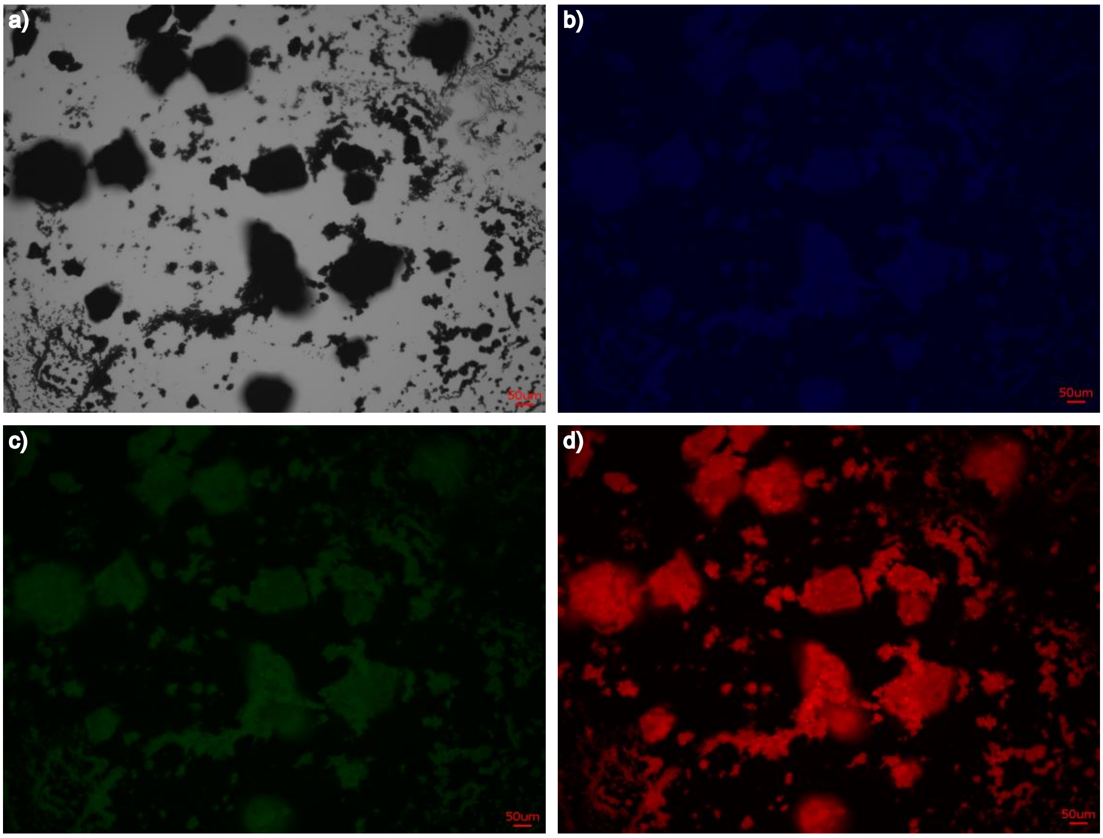

Figure S20: Staining of Indulin with AO (low) (light exposure time 200 ms); a) digital microscopic image, b) blue channel, c) green channel, d) red channel (scale bar 50 µm, image size 146 x 110 mm).

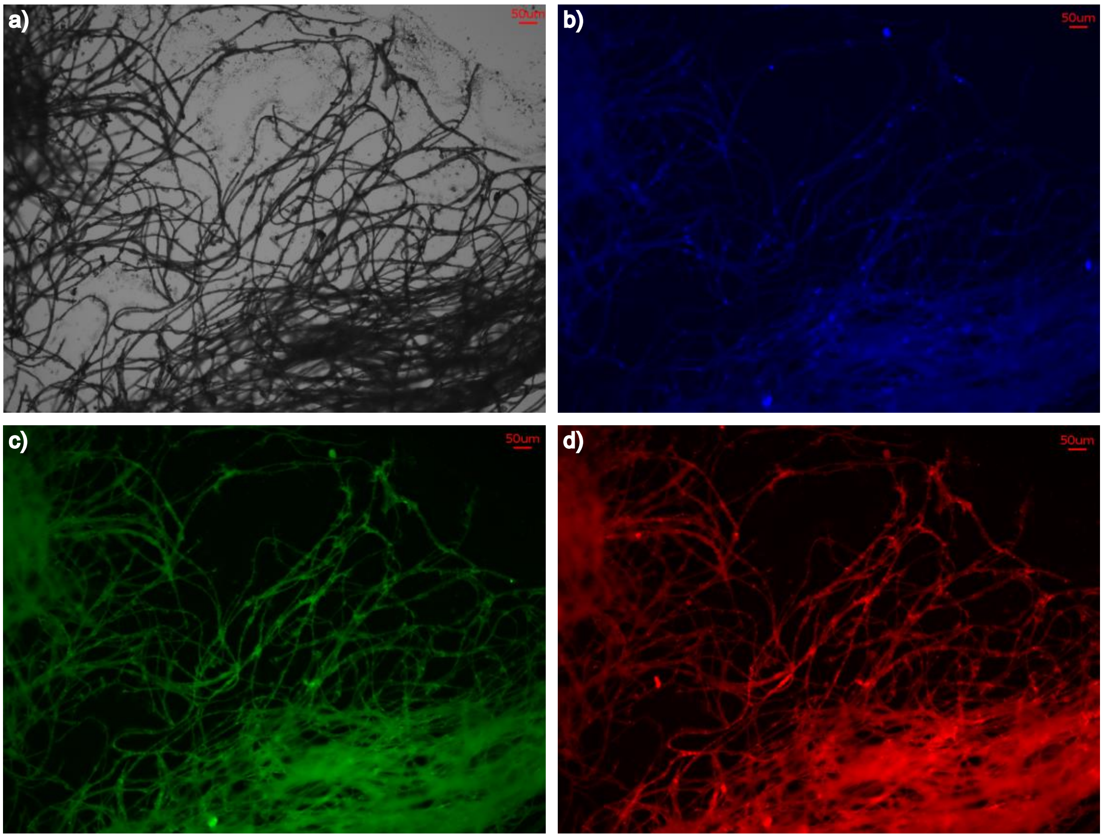

Figure S21: Staining of Stalk_raw with AO (low) (light exposure time 200 ms); a) digital microscopic image, b) blue channel, c) green channel, d) red channel (scale bar 50 µm, image size 146 x 110 mm).

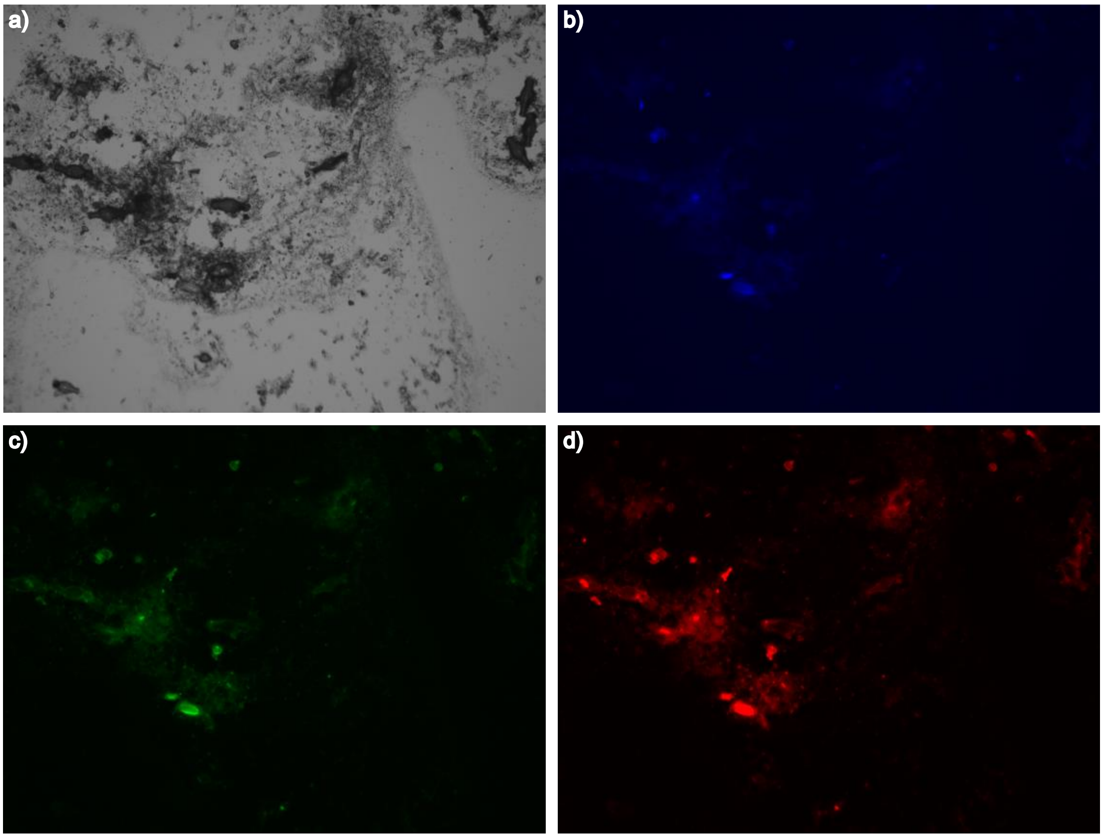

Figure S22: Staining of Stalk_EDTA with AO (low) (light exposure time 250 ms); a) digital microscopic image, b) blue channel, c) green channel, d) red channel (scale bar 50 µm, image size 146 x 110 mm).

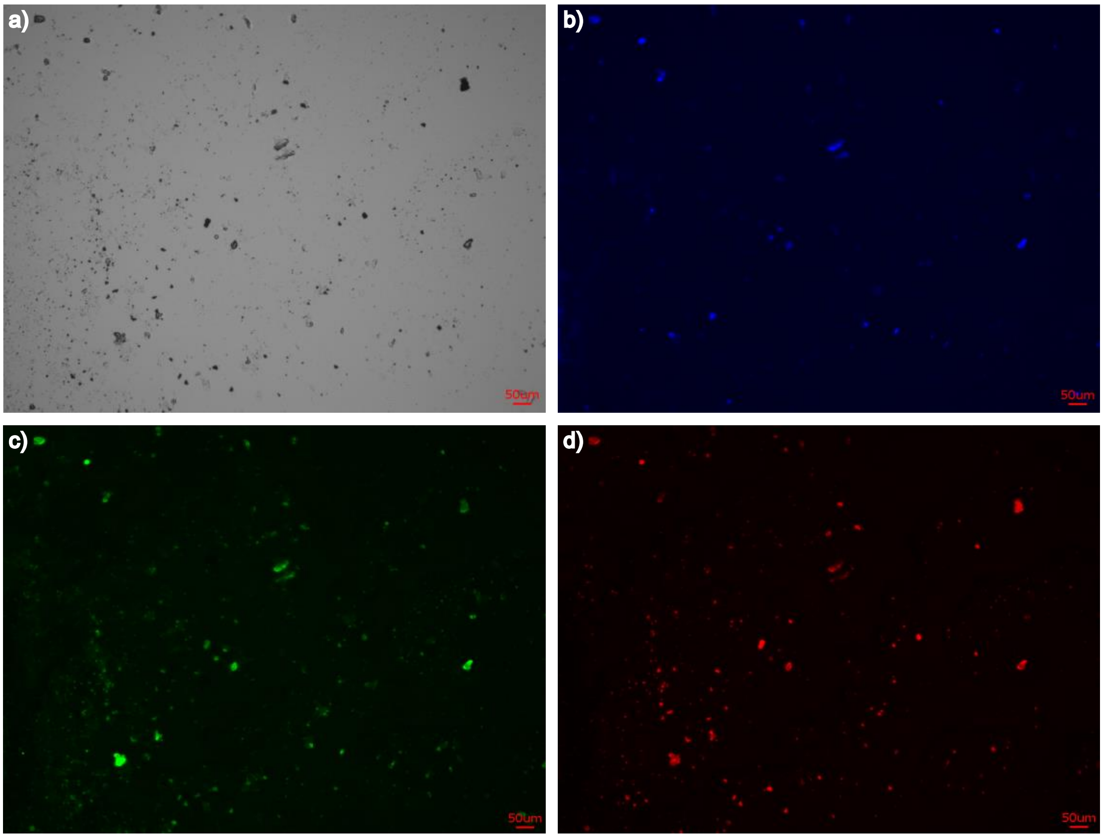

Figure S23: Staining of Indulin with AO (high) (light exposure time 250 ms); a) digital microscopic image, b) blue channel, c) green channel, d) red channel (scale bar 50 µm, image size 146 x 110 mm).

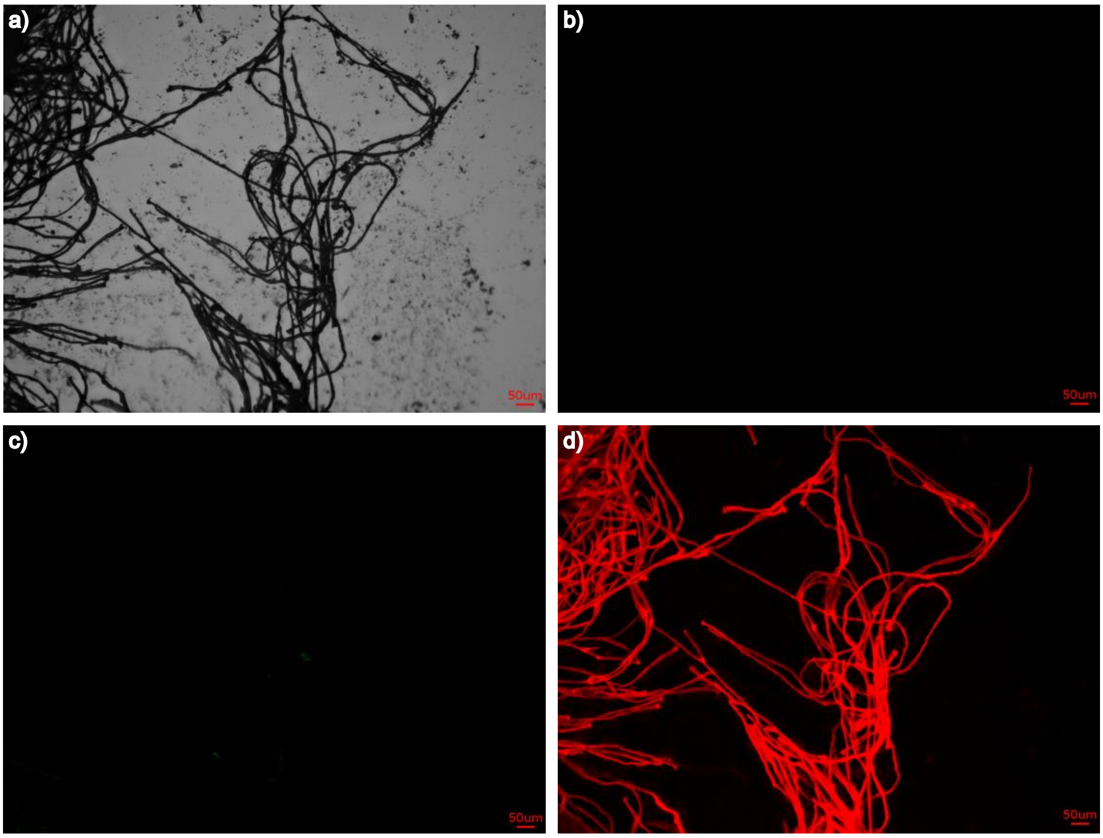

Figure S24: Staining of Stalk_raw with AO (high) (light exposure time 4.0 ms); a) digital microscopic image, b) blue channel, c) green channel, d) red channel (scale bar 50 µm, image size 146 x 110 mm).

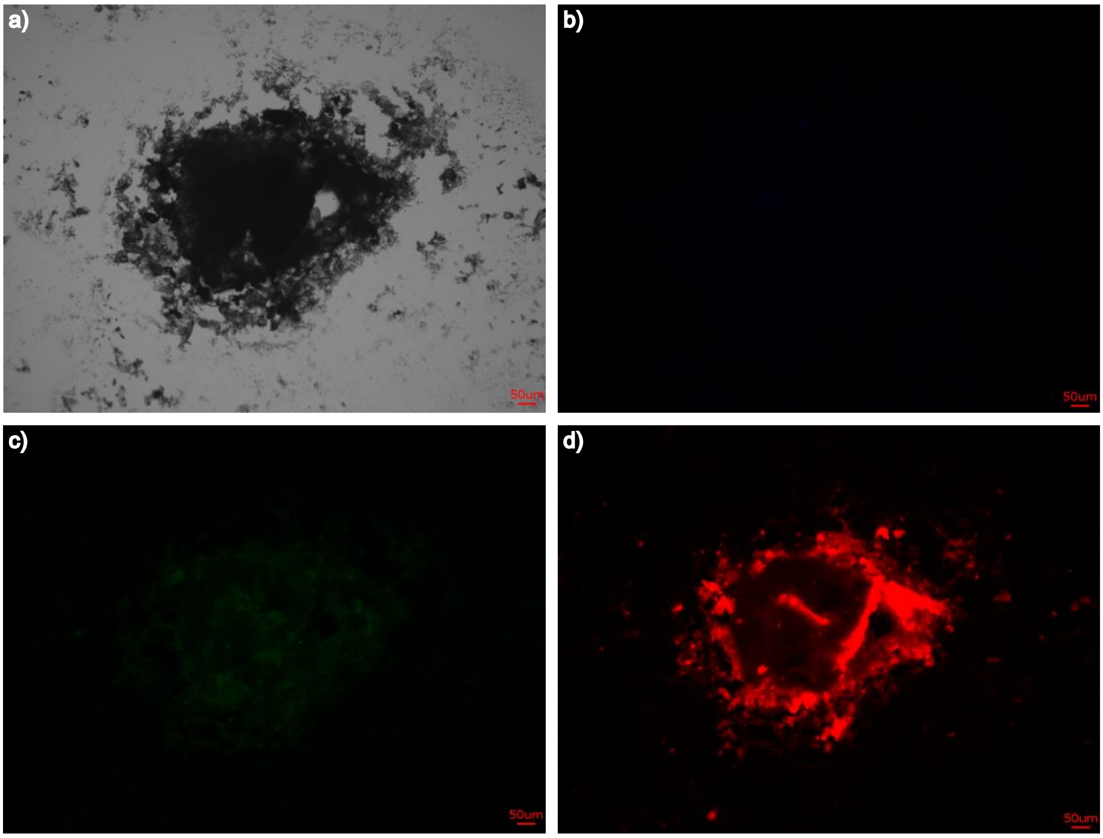

Figure S25: Staining of Stalk_EDTA with AO (high) (light exposure time 200 ms); a) digital microscopic image, b) blue channel, c) green channel, d) red channel (scale bar 50 µm, image size 146 x 110 mm).

References

[1] Lund A, Shiotani M, Shimada S. Principles and Applications of ESR Spectroscopy. Dordrecht: Springer Netherlands; 2011. https://doi.org/10.1007/978-1-4020-5344-3.

[2] Hofmeister AM, Rossman GR, Hofmeister AM, Rossman GR. Determination of Fe3+ and Fe2+ concentrations in feldspar by optical absorption and EPR spectroscopy. Phys. Chem. Minerals 1984; 11(5):213–24. https://doi.org/10.1007/BF00308136.

[3] Li K, Reeve DW. Fluorescent Labeling of Lignin in the Wood Pulp Fiber Wall. J. Wood Chem. Technol. 2005; 24(2):169–81. https://doi.org/10.1081/WCT-200026572.

[4] Houtman CJ, Kitin P, Houtman JCD, Hammel KE, Hunt CG. Acridine Orange Indicates Early Oxidation of Wood Cell Walls by Fungi. PLoS One 2016; 11(7):e0159715. https://doi.org/10.1371/journal.pone.0159715.
